# Morphogenic Modeling of Corrosion Reveals Complex Effects of Intermetallic Particles

**DOI:** 10.1002/advs.202404986

**Published:** 2024-08-19

**Authors:** Bruno C. Batista, Elena Romanovskaia, Valentin Romanovski, Michael Emmanuel, James T. Burns, Ji Ma, Istvan Z. Kiss, John R. Scully, Oliver Steinbock

**Affiliations:** ^1^ Department of Chemistry and Biochemistry Florida State University Tallahassee FL 32306 USA; ^2^ Department of Materials Science and Engineering Center for Electrochemical Science and Engineering University of Virginia Charlottesville VA 22904 USA; ^3^ Department of Chemistry Saint Louis University 3501 Laclede Ave. St. Louis MO 63103 USA

**Keywords:** alloy, corrosion, excitation waves, microstructure, nonequilibrium, self‐organization

## Abstract

Corrosion processes are often discussed as stochastic events. Here, it is shown that some of these seemingly random processes are not driven by nanoscopic fluctuations but rather by the spatial distribution of micrometer‐scale heterogeneities that trigger fast reactions associated with corrosion. Using a novel excitable reaction‐diffusion model, corrosion waves traveling over the metal surface and the associated material loss are described. This resulting nonuniform corrosion penetration, seen as a height loss in modeling, exposes buried intermetallic particles, which depending on the local electrochemical state of the surface trigger or block new waves. Informed by quantitative experimental data for the Mg–Al–Zn alloy AZ31B, wave speeds, wave widths, and average material loss are accurately captured. Morphogenic mitigation based on wave‐breaking microparticles is also simulated. While AZ31B corrosion is identified as a process driven by rare‐wave events, this study predicts several other corrosion regimes that proceed via spots or patchy patterns, opening the door for new protection, design, and prediction strategies.

## Graphical abstract

The corrosion of materials, driven by electrochemical waves, leads to localized material loss. This exposes buried intermetallic particles that are more reactive intermetallic particles (IMPs) and can trigger new waves. This destructive cycle is quantified for the important example of the Mg alloy AZ31B and a model is developed that accurately captures these observations. These findings open avenues for mitigating strategies and advanced alloy design.

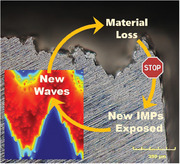

## 1 Introduction

Today's aerospace, automobile, and consumer industries require lightweight materials with high‐performance mechanical properties. These demands are at least partially met by magnesium alloys which are projected to reach a global market size of 6.6 billion US dollars by 2027.^[^ ^1^ ^]^ An even deeper utilization is currently hindered by the relatively low corrosion resistance of the alloys, which prompts broad and meaningful research targets for modern corrosion science.^[^ ^2^, ^3^, ^4^, ^5^, ^6^ ^]^ Of particular interest is the evaluation of the corrosion dynamics in terms of far‐from‐equilibrium patterns, traveling waves, and nonlinear electrochemistry along with morphogenic control and mitigation strategies.^[^ ^7^, ^8^ ^]^ This approach—compared to a compositional optimization and the application of coatings^[^ ^9^, ^10^, ^11^ ^]^—is relatively unexplored although it promises potentially profound technological and intellectual rewards.

Many electrochemical processes display time‐dependent oscillations, dual‐state reaction velocities, or natural variations in current or voltage within a certain spectrum of external factors.^[^ ^12^ ^]^ Spatial coupling, such as diffusion, tends to unfold the temporal dynamics over the electrode surface, inducing complex spatiotemporal patterns on meso‐ and macroscopic length scales.^[^ ^13^, ^14^, ^15^, ^16^ ^]^ Propagating waves are a typical example and were discovered 100 years ago in the context of “passive iron nerves” where dissolution fronts move with unusually high speeds of up to 1 m s^−1^.^[^ ^17^, ^18^ ^]^ Unlike electromagnetic and acoustic waves, these nonlinear waves have a constant amplitude and show no interference phenomena. On the contrary, their underlying energy dissipation and trailing refractory zones place them closer to phenomena such as spreading wildfires, falling domino chains, and traveling action potential in cardiac as well as neuronal tissue.^[^ ^19^, ^20^ ^]^

All of the latter examples belong to the class of excitable systems. In the absence of transport and spatial degrees of freedom, excitable behavior implies the existence of a stable steady state, that in response to a perturbation, has two qualitatively different recovery modes.^[^ ^19^ ^]^ While the response to small perturbations is unremarkable and monotonic, perturbations exceeding a system‐specific threshold perform a long excursion through phase space before returning to the steady state. In spatially extended systems, this response often creates traveling reaction zones, akin to those in electrochemical and corrosion systems. The classic example in chemistry is the Belousov‐Zhabotinsky reaction which self‐organizes wave patterns that emerge as blue bands from the chemically reduced, red background.^[^ ^21^ ^]^ Similar excitation waves also exist on catalyst surfaces such as Pt(100) catalyzing the oxidation of CO gas.^[^ ^22^, ^23^ ^]^ The presence of these moving reaction zones is driven by the diffusion of an activator, often an autocatalytic species, while the system recovery is orchestrated by a control/inhibitory species that creates a trailing refractory zone in which re‐excitation is impossible or more difficult.

Modern electrochemistry has identified such propagating corrosion waves on various metals and alloys.^[^ ^24^ ^]^ For high‐purity magnesium in seawater, corrosion waves travel with speeds of about 0.1 mm min^−1^, leading to material fatigue and failure.^[^ ^24^ ^]^ Magnesium dissolution waves are observed under potentiostatic and galvanostatic conditions, but—depending on the experimental settings—can be accompanied or replaced by other spatiotemporal phenomena such as filiform corrosion.^[^ ^25^ ^]^ Corrosion waves in this instance are maxima in local electrochemical current density arising from electron transfer as Mg is oxidized. While some waves can be qualitatively monitored by optical imaging, a preferred way to study wave dynamics is the scanning vibrating electrode technique (SVET) which yields time‐resolved maps of the local oxidation and reduction current densities affiliated with corrosion (Figure S1, Supporting Information).^[^ ^26^, ^27^, ^28^, ^29^, ^30^, ^31^ ^]^ Such measurements have shown that waves also exist on Mg alloys including AZ31B,^[^ ^32^, ^33^, ^34^, ^35^, ^36^ ^]^ and these waves will be the main focus of this report.

Like most alloys, AZ31B is not homogeneous but possesses an intricate grain structure, often with basal plane texturing, and large numbers of intermetallic particles (IMPs) that form during the production of the material.^[^ ^37^, ^38^, ^39^ ^]^ The IMPs are mainly Al‐Mn particles, such as Al_11_Mn_4_, Al_8_Mn_5_, and ɛ‐AlMn, and cathodically active with respect to the Mg matrix.^[^ ^35^, ^40^, ^41^ ^]^ These elements often have low solubility in Mg and are intentionally added in some cases. Intermetallic particles trigger local galvanic corrosion cells with the surrounding Mg, owing to their locally high electrode potentials and potential driving force, causing oxidation relative to the surrounding alloy matrix. For instance, under charge transfer control, the current density is exponential with overpotential. Based on substantial in‐situ and ex‐situ observations^[^ ^42^, ^43^, ^44^ ^]^ and the additional experimental evidence in Table S2 and Figures S2, S3 (Supporting Information), we therefore assert that these micrometer‐sized particles exposed on the corroding surface affect wave nucleation and propagation as well as the resulting corrosion rates and surface topographies. Another feature of Mg corrosion is anodically induced cathodic activation due to the surface accumulation of unoxidized transition metals.^[^ ^45^ ^,^ ^46^ ^]^ This occurs for many different Mg alloys.^[^ ^47^ ^]^ Furthermore, there is a negative difference effect when the matrix is anodically polarized corresponding to an unexpected increase in hydrogen evolution.^[^ ^48^, ^49^, ^50^, ^51^, ^52^, ^53^ ^]^ We also note that the detailed mechanism of Mg dissolution remains an active area of research.^[^ ^47^, ^54^ ^]^

While earlier studies reported models for different facets of Mg corrosion, the integration of these factors has remained challenging, primarily due to the intrinsic far‐from‐equilibrium and multiscale nature of the process.^[^ ^55^, ^56^, ^57^ ^]^ We hence start our analysis on the basis of a nonspecific reaction‐diffusion model capable of describing nonlinear waves while allowing for fast numerical simulations. Intellectually, this approach builds upon an idea first formulated for the stochastic description of the cooperative onset of high‐activity (pitting) corrosion for stainless steel^[^ ^58^ ^]^ while also drawing from models of intergranular corrosion along sensitized grain boundaries.^[^ ^59^, ^60^ ^]^

The specific goal of our study is to provide a semi‐empirical model of surface corrosion capable of describing wave propagation, the associated material loss, and the resulting increase in surface roughness. All wave initiations are triggered by surface‐exposed IMPs, an assumption supported by experimental data (Figure S3, Supporting Information). For the example of AZ31B, the model is calibrated based on experimentally determined wave speeds, pulse widths, and the average material loss per wave passage. Further informed by measured IMP size distributions and number densities, the resulting model is an effective platform for exploring the state‐dependent response of the system to IMPs, the testing of mitigation strategies, and possibly applications to other alloys functioning as sacrificial corrosion or battery anodes.

## 2 Results and Discussion

**Figure 1:**
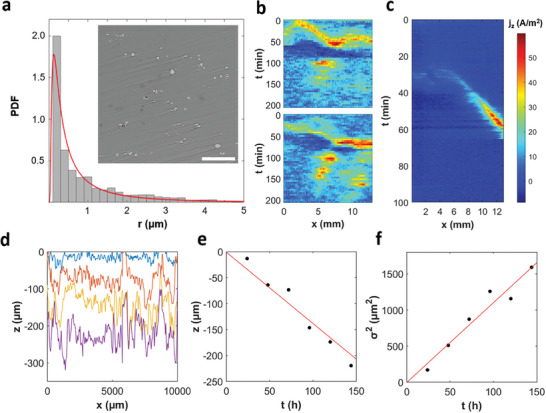
Experimental characterization of microstructure, corrosion waves, and surface damage for the Mg alloy AZ31B. a) Size distribution of intermetallic particles (IMPs, *N* = 1634). Inset: Backscatter electron image of a representative alloy surface. Scale bar: 100 µm. b,c) Time–space plots compiled from SVET‐derived current density maps of the alloy under 0.6 m NaCl(aq) reveal traveling waves and spots. In c), the profiles are oriented in the direction of wave propagation. d) Penetration depth profiles of the corroding alloy obtained after 24, 72, 96, and 144 h, where *z* = 0 is the uncorroded state. e,f) Average and variance of the surface height *z*(*x*) as a function of time, respectively. Continuous lines are best fits assuming proportional dependencies.

Heterogeneities, such as grain boundaries as well as nano and micro‐sized inclusions, greatly affect the corrosion rate of materials.^[^ ^38^, ^48^ ^]^ We therefore characterize the IMP distribution in AZ31B by backscatter electron imaging in scanning electron microscopy. The inset of **Figure** **1**a shows a representative micrograph in which IMPs are small bright regions of irregular and widely varying shape. Detailed image analyses (Figure S4, Supporting Information) yield an average number density of 880 IMPs mm^−2^ and a size distribution that is described by an inverse Gaussian function with a mean of *μ* = 0.909 µm and a shape parameter of *λ* = 0.385 µm (red curve in Figure 1a). The IMP locations show some spatial correlation, which is attributed to the existence of fractured particle lines extending along the alloy's rolling direction, formed during alloy production. Furthermore, considering the strongly negative standard oxidation/reduction potential of magnesium (*E*° = −2.37 V), we can conclude that most if not all IMPs play an activating role in AZ31B corrosion. A detailed characterization of the alloy and its IMP compositions are provided in Tables S1, S2 (Supporting Information), respectively.

In corrosive solutions, the exposed AZ31B surface develops self‐organized macroscopic patterns that we study for the example of 0.6 m NaCl(aq) to approximate real‐use conditions encountered in chloride‐containing environments. By visual inspection, these corroding areas can be discerned as darker regions, due to different white‐light reflectivities of Mg and Mg(OH)_2_ (Figure S2, Supporting Information). The patterns vary greatly over time but are overall reminiscent of either traveling waves or intermittent spots. They also produce sheet‐like streams of very small rising H_2_ bubbles that accompany the leading edges of the moving patterns (Movie S1, Supporting Information) as shown elsewhere.^[^ ^61^, ^62^ ^]^

We quantify these spatiotemporal patterns using SVET which resolves the local net *z*‐direction corrosion currents in both space and time, yielding heatmap videos of the corrosion activity expressed as net anodic and cathodic dissolution rates (Figure 1b). To summarize the spatiotemporal dynamics in a compact and informative way, we extract 1D profiles from these image sequences and stack them in a downward direction to capture the corrosion progression with respect to exposure time. Figure 1c shows a representative example of the resulting time‐space plots for AZ31B under open‐circuit conditions. The patterns manifest as regions of anodic activity (red) on a cathodic (blue) background. The waves appear as diagonal lines with slopes inversely proportional to their propagation speed. For the example in (c), the profile is carefully aligned in the perpendicular direction of the 2D front yielding a wave speed and anodic pulse width of 120 µm min^−1^ and 1 mm, respectively. Both quantities exhibit minimal variation from wave to wave and can be considered characteristic of the specified alloy and experimental conditions.

The local anodic current densities obtained from calibrated and *z*‐corrected SVET measurements also inform the determination of the material loss per wave event, an important reference point for our model. For this analysis, we integrate the current density over space and reference it to the Faraday law to obtain the number of moles oxidized. Further considering the alloy density, we thus estimate the average height loss per wave passage as 21 µm.

Our SVET results are complemented by profilometric measurements that monitor the corroding alloy surface under identical conditions but for different samples. Corroded samples are chemically cleaned prior to metrology measurements. Figure 1d presents height traces for successive corrosion times between 1 h and 6 d. These data yield the mean height and height variance as a function of time (Figure 1e,f). Both panels indicate a linear dependence allowing us to determine the average corrosion rate as 0.023 µm min^−1^ and the variance rate as 0.18 µm^2^ min^−1^. Notice that the variance is the square of the standard deviation and hence the square of the surface roughness.

The linear increase of the variance suggests that—at some empirical level—the material loss shares similarities with a simple random walk.^[^ ^63^ ^]^ Such a process based on the random height loss of a discrete surface is shown in Figure S5 (Supporting Information). This purely stochastic process yields a constant corrosion rate d*z*/d*t* and a variance that increases linearly with an increasing number of iterations. While these features match our experimental result, the surface profiles are pure white noise and thus differ from the experimental profiles in Figure 1d which are locally correlated and form distinct intervals of lower and elevated values. This simple discussion illustrates the ambiguity of corrosion^[^ ^13^ ^]^ in the light of random versus cooperative, deterministic processes.

**Figure 2:**
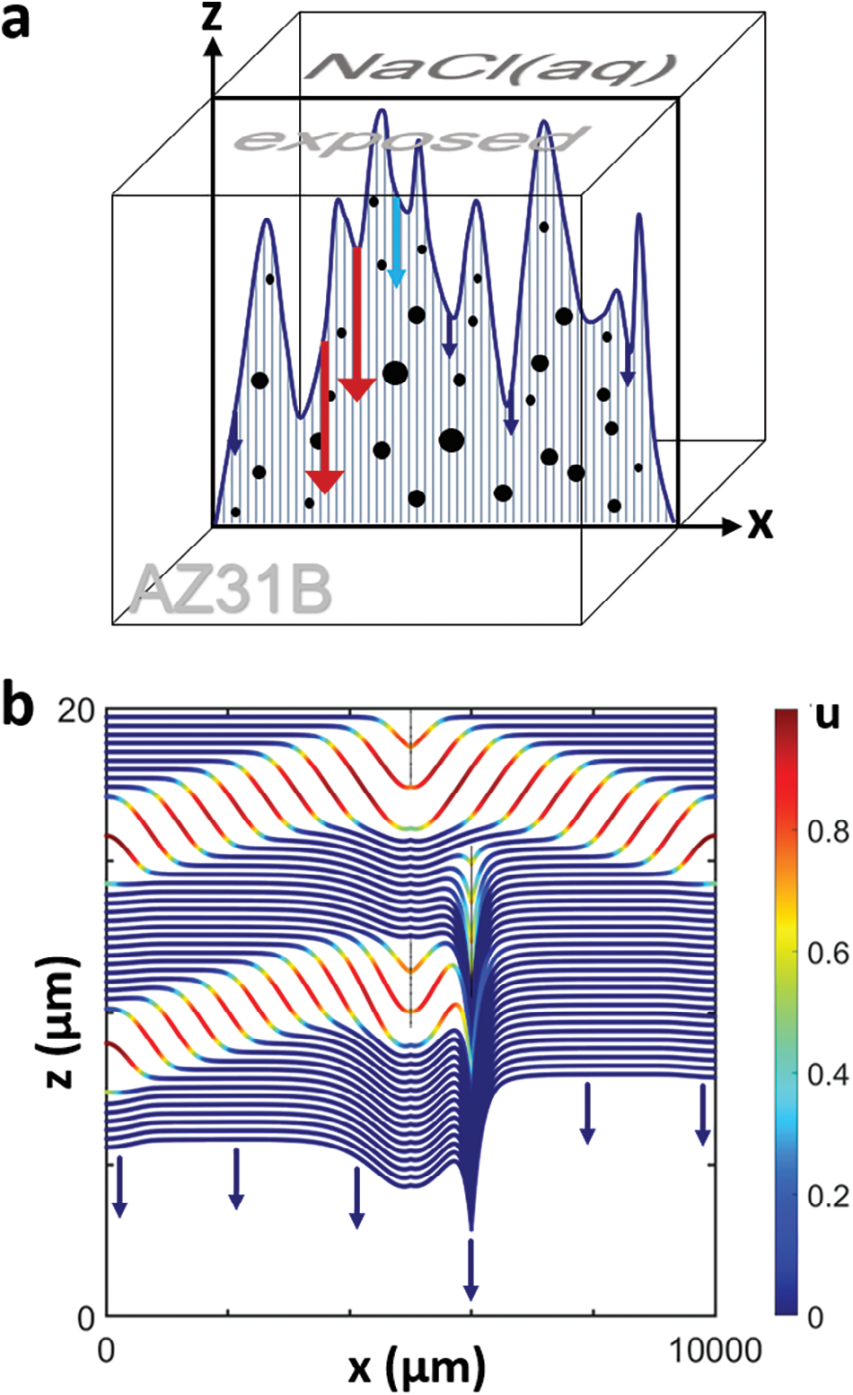
Schematic and model case simulation of wave‐induced damage morphology. a) Illustration of our model's focus on a single lamella normal to the alloy's surface. The sample corrodes in the downward direction with rates (arrows) that depend on the dynamic state of the interface and IMPs (black dots) being unearthed. b) Numerical simulation of the *z*(*x*) surface profiles sampled every 4 min. Colors indicate the local state of *u*(*x*) with red resulting in fast corrosion. Three circular IMPs appear as thin vertical lines due to the plot's extreme aspect ratio. The topmost IMP nucleates a propagating wave pair, while the second is inhibited by the earlier wave and causes a cusp‐like hole. The lowest IMP is partially inhibited and initiates a leftward moving wave pulse. Parameters: *ζ* = 0.071 µm min^−1^, *k* = 0.5 µm min^−1^, and *u*_IMP_ = 0.7.

To study this fundamental question in the context of dynamic systems and morphogenic processes, we now formulate a mathematical model that describes a single slice of the alloy perpendicular to the corroding surface. For this slice, the solution‐alloy interface is a 1D curve of height *z*(*x*) (**Figure** **2**a) that slowly moves downwards and roughens as the corrosion proceeds via waves and other patterns. The slice is populated with circular IMPs that match the experimentally determined size distribution and number density. Chemical differences and deviations from a uniform random distribution of the IMP locations are not considered yet, but will be discussed towards the end of this article. As we will show in the following, the exact locations of individual IMPs affect only the short‐term corrosion dynamics, but become less relevant over the long periods relevant for real‐world corrosion processes. These long‐term characteristics depend primarily on the IMP statistics.

The electrochemical dynamics at the moving solution‐alloy interface are modeled as an excitable system. As described in the Introduction, such systems perform a large‐amplitude pulse‐like response if perturbed above a certain threshold value before entering a refractory phase and finally returning to the steady state. This behavior is universal to all excitable systems, including certain electrochemical processes. We do not aim to formulate a detailed chemical mechanism but rather rely on this universality and its resulting spatiotemporal, morphogenic patterns. This simplified description of complex processes has proven successful in many biological contexts, ranging from cardiac arrhythmia.^[^ ^64^ ^]^ and tumor growth^[^ ^65^ ^]^ to neural networks.^[^ ^66^ ^]^ Nonetheless, the model to be described also serves as a versatile platform for the future integration of more detailed mechanistic aspects.

Our model suggests that corrosion spreads as a wave driven by the concentration of corrosive species like Cl^‐^, triggered by local film damage that attracts Cl^‐^ ions to maintain electroneutrality, accelerating corrosion through positive feedback.^[^ ^13^, ^67^ ^]^ It should be noted that both absence of Cl^‐^ and addition of chelators have strong effects in Mg experimental work.^[^ ^48^ ^]^ We construct our model around the Barkley equations^[^ ^68^, ^69^ ^]^ which describe the excitation behavior of a fast activator variable *u* (approximating the surface concentration of the corrosive species) and a slow control variable *v* (the surface recovery^[^ ^70^ ^]^). Both variables are dimensionless. To describe the material loss and resulting changes in the penetration depth *z*, a third equation is added yielding

(1) $$\frac{\partial u}{\partial t}=\nabla^{2}u+\frac{1}{\varepsilon }u\left(1-u\right)\left(u-\frac{v+b}{a}\right)$$

(2) $$\frac{\partial v}{\partial t}=u-v$$

(3) $$\frac{\partial z}{\partial t}=-\zeta -ku$$

The constants *ε* = 0.02, *a* = 0.44, and *b* = 0.045 create an excitable system capable of sustaining traveling waves (Figures S6, S7, Supporting Information), while the parameters *ζ* and *k* control the rate of background corrosion and the strength of *u*‐depending material loss, respectively.Space and time are scaled to match the experimentally observed wave speed and pulse width (Figure S7, Supporting Information). In addition, we assume that IMPs exposed to the surface curve, locally set the *u* variable to a constant value of *u*_IMP_ and that IMPs can be disregarded after the interface passes them. As the stable steady state of the Barkley system is (*u*,*v*) = (0,0), this local perturbation can—depending on the size of the particle and the state of the surrounding system—trigger corrosion waves with the necessary but not sufficient condition that *u*_IMP_ exceeds the excitation threshold *b*/*a*.

Figure 2b illustrates the effects of three deliberately placed IMPs on the corrosion of an initially flat alloy surface at *z* = 20 µm. It shows the surface's evolution over time, with red indicating areas of intense corrosion and blue indicating slower, background corrosion. The first IMP, located at *x* = 5000 µm, generates two corrosion fronts moving in opposite directions. The background corrosion, represented by a series of parallel blue curves, progresses slowly compared to the faster material degradation in the red areas due to a higher value of the *k u* term in Equation 3.

The second IMP, at *x* = 6000 µm and 6 µm below the surface, is larger but fails to initiate corrosion fronts due to the surface's temporary refractoriness (high *v* value) caused by the previous corrosion wave, yet it forms a sharp, pit‐like defect. The third IMP, at *x* = 5000 µm and 1 µm deeper than the second, triggers a corrosion wave moving leftward while the rightward moving front is suppressed by the effect of the second IMP. This example illustrates the complex interactions between IMPs and the alloy surface, showing that IMPs can both initiate corrosion waves and block them or form microscopic hotspots, contributing to the surface roughness. These processes appear random but are fully deterministic, with the corrosion pattern and the exposure rate of IMPs being determined by their positions and the initial surface state.

**Figure 3:**
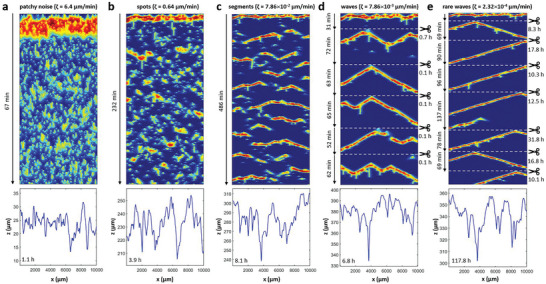
Different corrosion regimes in our model for decreasing background corrosion rates *ζ*. The top panels are time–space plots showing the temporal evolution of *u*(*x*) with time evolving in the downward direction. The columns illustrate the corrosion regimes a) patchy noise, b) spots, c) segments, d) waves, and e) rare waves. Scissors and dashed lines indicate that segments of specified durations are omitted as max(*u*) <10^−5^. Color coding as in Figure 2b. The lower panels show the surface profiles for the specified times which correspond to the last line in the time‐space plot above. The simulations use the same IMP map. Parameters: *k* = 3 µm min^−1^, *u*_IMP_ = 0.6, and *ζ* as indicated above each column.

**Figure** **3** shows examples of the corrosion dynamics in a system with IMPs that match the experimentally determined number density and size distribution. The top row are time‐space plots that recapitulate the evolution of the model variable *u*(*x*,*t*) for a 1 cm long sample and times between about 1 h and 5 d. Time increases in the downward direction and, as before, blue and red colors indicate weak and strong corrosion activity, respectively. The scissor symbols signify the removal of long quiescent periods with the respective time interval specified below the symbol. Below each time‐space plot is a graph showing the surface topography at the end of the respective simulation. The columns differ only in the rate of background corrosion *ζ*. The results in Figure 3a–e reveal distinct corrosion regimes that we coin patchy noise, spots, segments, waves, and rare waves, respectively. These regimes occur with decreasing *ζ* values that here are varied between 6.4 µm min^−1^ (a) and 2.3 × 10^−4^ µm min^−1^ (e). The transitions between the different regimes are continuous and hence no transition points can be specified.

For rare waves (column e), we find very long quiescent time intervals between waves that can exceed 1 day. These time intervals vary substantially between wave events and reflect the distance of the current surface to the nearest IMP. Once this IMP is exposed, it tends to initiate a wave traveling in both directions. As the background corrosion rate *ζ* is increased, the average waiting times decrease. Around *ζ* = 8 × 10^−3^ µm min^−1^ (d), we often find several consecutive waves without intermittent quiescent periods. For values around *ζ* = 8 × 10^−2^ µm min^−1^ (c), this faster sequence of wave initiations leads to wave blocks that arise from local refractory zones generated primarily by the preceding wave. These annihilation events create wave segments of shorter reach. For even faster background corrosion, the segments evolve into spots and ultimately patchy noise patterns.

All corresponding surface profiles *z*(*x*) appear erratic with broader features possibly occurring for wave‐based corrosion (c–e). Notice that the unperturbed passage of a wave through the entire medium causes a substantial loss of material but does not contribute to an increase in surface roughness. The roughness increases primarily via blocked waves and segments as well as IMPs modulating the amplitude of the passing corrosion front. We attempted to approximate these profiles by integrating the IMP maps (blurred by Gaussian functions) in the *z*‐direction (Figure S8, Supporting Information). While the results showed some qualitative similarities, they were not sufficient to predict the observed *z*(*x*) curves re‐emphasizing the importance of the interplay between wave patterns, IMP locations, and state‐dependent material loss.

In additional simulations, we evaluated the surface dynamics in the absence of background corrosion (*ζ* = 0). This scenario is interesting as the system cannot unearth new IMPs unless waves are present. As expected, corrosion stalls quickly after the passage of a first wave pattern and then never reignites. However, for sufficiently large *k* values (Figure S9, Supporting Information), we identify an additional regime, coined “leapfrogging”, for which IMPs create low‐amplitude corrosion spots and short segments that hop from IMP to IMP, thus causing a nearly steady loss in material. For the IMP number density of AZ31B, the overall corrosion process is very fast and for *k* = 150 µm min^−1^ causes 1 cm deep corrosion damage in 4.2 d.

We emphasize that in our simulations both the average height loss and the surface variance increase linearly with the elapsed corrosion time. An example for rare waves extending over 82 d is shown in Figure S10 (Supporting Information). These findings are in agreement with the experimental observations in Figure 1e,f and also capture the overall noisier character of the roughness data. Notice that—accelerated by an analytical integration during quiescent time intervals (see Experimental Section)—these 82 d of corrosion dynamics can be computed on a personal computer in two days, yielding a speed‐up factor of about 40 when comparing the simulation to the real‐world process.

**Figure 4:**
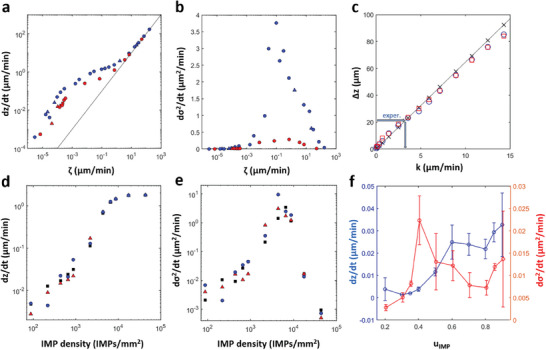
Material loss and build‐up of surface roughness in our model. a) Double‐logarithmic plot of the average height loss d*z*/d*t* as a function of the background corrosion parameter *ζ*. The continuous line indicates a power law exponent of 1. b) Rate of the surface variance *σ* ^2^ increase as a function of the background corrosion parameter *ζ*. a,b) Red and blue markers correspond to *k* = 3 and 10 µm min^−1^, respectively. Different symbols distinguish between different random realizations of the used IMP maps. c) Surface loss Δ*z* after the passage of one wave in a system with no IMPs as a function of the model parameter *k* (x‐shaped markers) and the best‐fit proportional dependence (line). Squares and circles are loss values from a single wave in the presence of IMPs with two different random distributions. d) Average height loss and e) rate of surface variance as a function of the number density of intermetallic particles. f) Average height loss d*z*/d*t* (blue) and variance rate (red) as a function of the IMP strength *u*_IMP_. Error bars indicate the standard deviation for *N =* 3–6 simulations with different random IMP maps. a–c) *u*_IMP_ = 0.6, c,d) *ζ* = 3 × 10^−4^ µm min^−1^, and d) *k* = 3 µm min^−1^.

Next, we investigate the dependence of both the average material loss rate d*z*/d*t* and the surface roughening rate d*σ* ^2^/d*t* as a function of the key model parameters *k* and *ζ* (**Figure** **4**a,b). The first step of this analysis is to match, if possible, the experimentally observed height loss of 21 µm per wave, average loss rate of 0.023 µm min^−1^, and roughening rate of 0.18 µm^2^ min^−1^ (see Figure 1) under the already applied constraints of a realistic IMP distribution, wave speed, and wave width. The primary goal is then to assign the correct corrosion regime (Figure 3) to the NaCl(aq)‐exposed AZ31B.

Figure 4c shows the *k*‐dependence of the damage Δ*z* caused by the passage of a single corrosion wave through the IMP‐free alloy (x‐shaped markers). This linear curve is essentially independent of the background corrosion rate *ζ* as long as the respective material loss is very small during the time *τ*_L_ = *L*/*v* needed for the wave to traverse the system length *L* (*v* = 120 µm min^−1^ as noted earlier). For larger *ζ* values, the curve shifts upwards by *ζ τ*_L_. From the experimental target value of 21 µm per wave and the linear curve in Figure 4c, we obtain good agreement for *k* = 3 µm min^−1^. Notice that this estimate is nearly unchanged if the wave propagates in systems with IMPs (circles and squares in Figure 4c).

The average corrosion rate d*z*/d*t* increases with increasing background corrosion rates *ζ*. A double‐logarithmic plot (Figure 4a) reveals a transition between two power laws. For large *ζ*, the power‐law exponent is 1 indicating a linear dependence that is unaffected by the patterns on the corroding alloy and solely dependent on the rapid background corrosion. For small *ζ*, the power law exponent appears to be slightly larger than 1 and the data are *k*‐dependent. Notice that red markers in Figure 4a correspond to the value *k* = 3 µm min^−1^ that we determined from the data in Figure 4c. For these simulation results, the experimentally measured corrosion rate of 0.02 µm min^−1^ is reproduced for *ζ* ≈ 2.32 × 10^−4^ µm min^−1^ which implies that AZ31B corrosion in 0.6 M NaCl solution obeys the characteristics of rare‐wave dynamics (Figure 3e).

The same set of simulations also reveals insights into the evolution of the surface roughness *σ*. As noted above, our experimental and numerical results suggest a linear increase of the variance *σ* ^2^. Figure 4b graphs the corresponding average rate d*σ* ^2^/d*t* as a function of *ζ*. This rate is low for both very small and very large *ζ* values peaking around *ζ* = 0.1 µm min^−1^. The low roughening rate for large *ζ* is easily explained by the strong dominance of the background corrosion rate over wave‐ and IMP‐dependent material loss. The behavior for low *ζ* reflects the very long quiescent times in the rare wave regime.

The average number of intermetallic particles exposed on the surface plays a crucial role in the evolution of surface height and roughness. Figure 4d shows that the rate of height loss increases proportionally to the surface density of IMPs, up to a maximum value of 50 µm min^−1^. Figure 4e reveals that the surface variance passes through a maximum at 8000 IMPs mm^−2^.

Another important factor is the activating strength of the IMPs, which is determined by the IMP compositions and resulting potential differences with respect to the surrounding bulk phase. Notice that up to this point, we have modeled IMPs as small heterogeneities with *u* = *u*_IMP_ = 0.6. Figure 4f shows the effect of a broader range of *u*_IMP_ values on the average corrosion rate (blue circles) and the roughening rate (red triangles) as obtained for the experimentally matched values of *k* = 3 µm min^−1^ and *ζ* = 2.32 × 10^−4^ µm min^−1^. Very small values (*u*_IMP_ < 0.2) fail to initiate waves as expected from the system's excitation threshold of *b*/*a* = 0.1. For larger values, the corrosion rate increases somewhat linearly with *u*_IMP_ due to more severe local damage and possibly more frequent wave initiations. Furthermore, we find that the surface roughness is largest around *u*_IMP_ = 0.4 for which d*σ* ^2^/d*t* is about twice as large as for the earlier used value of *u*_IMP_ = 0.6. Despite this possible enhancement of the roughening rate, our simulations still fall short by a factor of about 5 compared to the experimentally observed value of 0.18 µm^2^ min^−1^ (see Figure 1).

For both experiments and simulations, a systematic and reliable characterization of the surface roughening rate is difficult as this measure is prone to temporal and sample‐specific variations. Nonetheless, the difference by a factor of 5 is sufficient to require further discussions. One possible source of the discrepancy is the presence of spatial correlations in the positions of the IMPs. In many rolled alloys, such as AZ31B, IMPs tend to show some degree of clustering and even strings^[^ ^71^ ^]^ of fractured particles in the rolling direction (Figures S11, S12, Supporting Information). To capture this feature, we developed a semiquantitative method for reproducing these stringers that honors the experimentally determined particle size distribution and number density (Figure S12, Supporting Information, right column). This method randomly assigns center points of the clusters and then randomly arranges, within preset limits, IMPs along lines of different lengths around these points (see Supporting Information for details). These spatially correlated “stringer” maps are then compared to otherwise identical simulations with uncorrelated IMP maps. The results are shown in Figure S13 (Supporting Information) but reveal no statistically significant difference.

**Figure 5:**
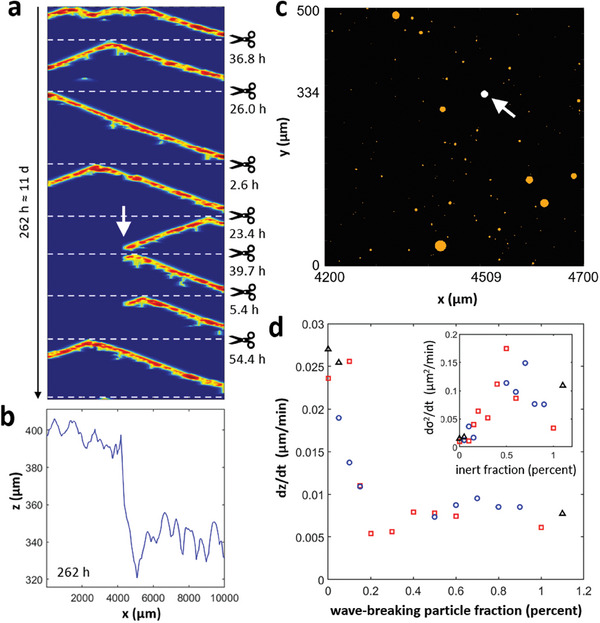
Simulated corrosion in the presence of wave‐breaking particles. a) Time‐space plot of corrosion waves affected by a wave‐breaking particle (white arrow) and b) resulting surface profile. c) Magnified view of the spatially uncorrelated IMP map used for the simulation in (a,b). Orange particles are activating, whereas the white particle caused the propagation failure indicated by the white arrow in (a). d) Average corrosion rate as a function of the percentage of wave‐breaking particles. The inset shows the corresponding rate of the increasing surface variance. Symbols distinguish different random IMP maps generated for the same statistics. Model parameters: *ζ* = 2.32 × 10^−4^ µm min^−1^, *k* = 3 µm min^−1^, *u*_IMP_ = 0.6, and wave‐breaking particle fraction *ξ* = 0.4% (a–c).

Another explanation for the rapid increase in the surface roughness of corroding AZ31B is the presence of wave‐breaking microparticles. The time‐space plot in **Figure** **5**a shows wave patterns during an 11 d period simulated using optimal model parameters and spatially uncorrelated IMP maps that follow the measured size distribution and number density. After about 5 days, an inert particle is exposed (white arrow) that blocks three leftward moving waves prior to falling off the simulated surface line. These wave blocks greatly affect the resulting surface profile *z*(*x*) (Figure 5b), which shows a step‐like difference of about 50 µm between the less corroded left and more damaged right side. Notice that the height change occurs at the wave‐breaking particle position (*x* = 4509 µm), which is shown along with its closer neighborhood of activating particles in Figure 5c.

The effect of wave‐breaking particles on the corrosion characteristics can be profound even if they constitute fewer than 1% of all microparticles in the alloy. Figure 5d shows the average corrosion rate as a function of the wave‐breaking particle fraction based on three IMP maps generated from the same distribution parameters (distinguished by different markers). We find a steep decrease of about 80% around particle fractions of about 0.2% which is accompanied by an increase in the roughening rate that for fractions above 0.5% slowly decreases again (see inset of Figure 5f). This increase in the surface roughness for very low inert particle fractions is a likely explanation for the lower roughening rates in our simulations with strictly activating particles (Figure 4b).

In addition to resolving the last mismatch between simulations and experiments, the results in Figure 5f suggest possible mitigation strategies for AZ31B and similar alloys with surface‐bound corrosion waves. One can envision a deliberate addition of inert particles, such as ceramics, either during the late stages of alloy production or during a post‐production surface treatment. These wave‐breakers would block waves and thus reduce the average corrosion rate. Our simulations presented here only considered a line across the 2D solution‐exposed surface and we anticipate that the effectiveness of wave‐breakers in two space dimensions will be reduced as the wave can move around the introduced obstacles. However, preliminary experiments and simulations strongly suggest that for AZ31B corrosion waves break up past the wave‐breakerS5 and thus preserve some significant degree of effectiveness.

## 3 Conclusion

In conclusion, our study on the magnesium alloy AZ31B suggests the existence of a cycle driven by intermetallic particle (IMP)‐based wave initiation, wave‐induced material loss, and the subsequent exposure of new wave‐initiating IMPs. Our semiempirical reaction‐diffusion model captures the fast surface dynamics (wave speed and width) as well as the underlying IMP distribution, tailored to AZ31B's behavior in salt water. Furthermore, the model replicates experimental observations like corrosion rate and surface roughness. Although our work focused on a specific alloy, it lays the groundwork for identifying similar processes in other materials; however, its applicability is currently limited to types of corrosion that result from surface dynamics rather than three‐dimensionally penetrating processes such as intergranular corrosion. Notably, our model can simulate weeks of corrosion in less than a day on a personal computer, offering practical insights for real‐world scenarios. Additionally, we showed that corrosion's perceived randomness is deterministic, driven by the locations of micrometer‐sized IMPs and their interactions via morphogenic patterns, with the main source of variability being the IMPs' nucleation and growth during alloy production.

## 4 Experimental Section

### Numerical Methods

Numerical simulations were performed in MATLAB R2020b by forward Euler integration on 5000 or 10 000 grid points spaced at Δ*x* = 1 µm, yielding system lengths of 5 or 10 mm, respectively. The small Δ*x* was needed to capture the size scales of the IMPs but necessitates very small time steps (here Δ*t* = 1.4 × 10^−5^ min). The domain was terminated by no‐flux boundaries. To allow for simulations over very long corrosion times, analytical solutions of the model were employed whenever max(*u*) < 10^−5^. These fast computation steps decreased *z*(*x*) linearly and *v*(*x*) exponentially, as given for *u* = 0, and terminated when *z*(*x*) reached the next IMP. In addition, Equation 1 and 2 were scaled with the help of the measured pulse width (1 mm) and wave speed (120 µm min^−1^) (see Figure S7, Supporting Information for additional details). The initial condition in all simulations was (*u*, *v*, *z*) = (0, 0, *z*_0_), where *z*_0_ is the initial sample height (typically 20 or 500 µm).

### Experimental Methods—Sample Preparation for Analysis

Samples were cut from a bulk piece of AZ31B‐H24. The ST surface was selected for analysis. All samples were polished using silicon carbide paper with a grit down to 1200. Then polished samples were sonicated in deionized water (18.2 MΩ cm) and isopropanol for 3 min before experiments. For SVET analysis, samples were molded into epoxy to fit the SVET electrochemical cell.

### Experimental Methods—IMP Characterization

The IMP number density, size distribution, and compositional distribution were analyzed by a dual‐beam scanning electron microscope (SEM) Helios UC G4 (Thermo Fisher Scientific, USA), energy dispersive X‐ray spectroscopy, and a Themis 60‐300 kV transmission electron microscope (TEM) (Thermo Fisher Scientific, USA) for 1 × 1 or 2 × 2 cm^2^ samples. Details are provided in the Supporting Information.

### Experimental Methods—Scanning Vibration Electrode Technique (SVET)

A Biologic M470 SP instrument was utilized for all SVET experiments. Corrosion experiments were conducted under open circuit potential (freely corroding) conditions. Exposed specimen surfaces were submerged completely in an electrolyte bath containing an aqueous 0.6 M NaCl solution for every experiment. The sample for the time‐space plot construction was 1.28 cm × 0.1 cm and scanned for 3.5 h. The sample with an area of 1.28 cm × 2.0 cm was utilized for the corrosion damage depth evaluation experiments. The platinum probe used in the vibrating SVET probe had a diameter of about 50 µm with a scan resolution of 100–200 µm, as indicated by the manufacturer. A detailed overview of the SVET instrument's design and calibration process can be found in previous papers.^[^ ^24^, ^72^ ^]^ The SVET probe was located vertically above the specimen test area and scanned at a constant height of 50 µm with an amplitude of 30 µm and a frequency of 80 Hz (except for the Au current source calibration experiment which is described in the Supporting Information). The peak‐to‐peak SVET voltage signal (*V*_pp_) is related to the current flux density along the axis of probe vibration (*j*_z_) by *V*_pp_ = *j*_z_ (*A*_pp_/*κ*), where *κ* is the solution conductivity and *A*_pp_ is the peak‐to‐peak amplitude of probe vibration, such that a quantity *G* = *κ*/*A*_pp_ is defined as the SVET calibration factor.

### Experimental Methods—Profilometry Measurements

The corroded surface morphology was investigated by 3D microscopy (Hirox RH‐8800, Hirox USA Inc.) under a magnification of ×150 with a detection limit of 1 µm^2^. The analyzed surface area was 1 cm^2^ for each sample.

## Conflict of Interest

The authors declare no conflict of interest.

## Author Contributions

E.R. and V.R. contributed equally to this work. Conceptualization and funding acquisition: J.B., I.K., J.M., J.S., O.S.; Experimental investigation: E.R., V.R., J.S.; Theoretical investigation: B.B., M.E., I.K., O.S.; Software: B.B., O.S.; Writing‐original draft: B.B., O.S.; Writing‐review & editing: B.B., I.K., E.R., V.R., J.S., O.S.

## Supporting information

Supporting Information

Supplemental Movie 1

## Data Availability

The data that support the findings of this study are available from the corresponding author upon reasonable request.

## References

[1] https://www.grandviewresearch.com/industry‐analysis/magnesium‐alloys‐market (accessed: May 2024).

[2] S. Sen‐Britain , S. Cho , S. Kang , Z. Qi , S. Khairallah , D. Rosas , V. Som , T. T. Li , S. Roger Qiu , Y. Morris Wang , B. C. Wood , T. Voisin , Nat. Commun. 2024, 15, 867. 38287015 10.1038/s41467-024-45120-6 PMC10825210

[3] H. Zhao , Y. Yin , Y. Wu , S. Zhang , A M. Mingers , D. Ponge , B. Gault , M. Rohwerder , D. Raabe , Nat. Commun. 2024, 15, 561. 38228660 10.1038/s41467-024-44802-5 PMC10792079

[4] Y. Xie , Nat. Mater. 2021, 20, 789. 33526878 10.1038/s41563-021-00920-9

[5] M. Stefanoni , U. M. Angst , B. Elsener , Nat. Mater. 2019, 18, 942. 31358940 10.1038/s41563-019-0439-8

[6] X. Guo , S. Gin , P. Lei , T. Yao , H. Liu , D K. Schreiber , D. Ngo , G. Viswanathan , T. Li , S H. Kim , J D. Vienna , J V. Ryan , J. Du , J. Lian , G. S. Frankel , Nat. Mater. 2020, 19, 310. 31988512 10.1038/s41563-019-0579-x

[7] B. Zaghloul , C. F. Glover , J. R. Scully , J. R. Kish , Corrosion 2021, 77, 192.

[8] Y. Yang , W. Zhou , S. Yin , S Y. Wang , Q. Yu , M J. Olszta , Y.‐Q. Zhang , S E. Zeltmann , M. Li , M. Jin , D K. Schreiber , J. Ciston , M. C. Scott , J R. Scully , R O. Ritchie , M. Asta , Ju Li , M P. Short , A M. Minor , Nat. Commun. 2023, 14, 988. 36813779 10.1038/s41467-023-36588-9 PMC9946947

[9] D. Wang , P. Zhou , Y. Zhang , T. Zhang , F. Wang , Corros. Sci. 2023, 222, 111428.

[10] M. Hu , Q. Tan , R. Knibbe , M. Xu , B. Jiang , S. Wang , X. Li , M.‐X. Zhang , Mater. Sci. Eng., R 2023, 155, 100746.

[11] C. Guo , Y. Li , C. Qi , H. Sun , Y. Xue , Y. Wan , D. Zhang , Surf. Coat. Technol. 2024, 476, 130209.

[12] K. Krischer , Adv. Electrochem. Sci. Eng. 2002, 8, 89.

[13] A. S. Mikhailov , S. Jain , L. Organ , J. L. Hudson , Chaos 2006, 16, 037104. 17014238 10.1063/1.2214155

[14] E. Nijholt , J. L. Ocampo‐Espindola , D. Eroglu , I. Z. Kiss , T. Pereira , Nat. Commun. 2022, 13, 4849. 35977934 10.1038/s41467-022-32282-4 PMC9385626

[15] K. Agladze , S. Thouvenel‐Romans , O. Steinbock , Phys. Chem. Chem. Phys. 2001, 3, 1326.

[16] F. Brau , S. Thouvenel‐Romans , O. Steinbock , S. S. S. Cardoso , J. H. E. Cartwright , Soft Matter 2019, 15, 803. 30644940 10.1039/c8sm01928b

[17] R. S. Lillie , J. Gen. Physiol. 1925, 7, 473. 19872151 10.1085/jgp.7.4.473 PMC2140733

[18] K. Agladze , O. Steinbock , J. Phys. Chem. A 2000, 104, 9816.

[19] A. T. Winfree , The Geometry of Biological Time, Springer, Berlin 2000.

[20] C. Beta , L. Edelstein‐Keshet , N. Gov , A. Yochelis , eLife 2023, 12, e87181. 37428017 10.7554/eLife.87181 PMC10332813

[21] R. H. Clarke , Nature 1970, 225, 535. 10.1038/225535a0 16056596

[22] S. Jakubith , H. H. Rotermund , W. Engel , A. von Oertzen , G. Ertl , Phys. Rev. Lett. 1990, 65, 3013. 10042757 10.1103/PhysRevLett.65.3013

[23] G. Ertl , Angew. Chem., Int. Ed. 2008, 47, 3524. 10.1002/anie.200800480 18357601

[24] G. Williams , H. N. McMurray , J. Electrochem. Soc. 2008, 155, C340.

[25] C. Kousis , P. Keil , H. N. McMurray , G. Williams , Corros. Sci. 2022, 206, 110477.

[26] H. S. Isaacs , A. J. Davenport , A. Shipley , J. Electrochem. Soc. 1991, 138, 390.

[27] F. Thébault , B. Vuillemin , R. Oltra , K. Ogle , C. Allely , Electrochim. Acta 2008, 53, 5226.

[28] A. M. Simões , J. Torres , R. Picciochi , J. C. S. Fernandes , Electrochim. Acta 2009, 54, 3857.

[29] A. C. Bastos , M. G. Ferreira , A. M. Simões , Corros. Sci. 2006, 48, 1500.

[30] M. J. Franklin , D. C. White , H. S. Isaacs , Corros. Sci. 1992, 33, 251.

[31] G. Williams , A. J. Coleman , H. N. McMurray , Electrochim. Acta 2010, 55, 5947.

[32] M. A. Melia , T. W. Cain , B. F. Briglia , J. R. Scully , J. M. Fitz‐Gerald , J. Magnesium Alloys 2017, 69, 2322.

[33] D. Thirumalaikumarasamy , K. Shanmugam , V. Balasubramanian , J. Magnesium Alloys 2014, 2, 36.

[34] S. Fajardo , G. S. Frankel , Electrochem. Commun. 2017, 84, 36.

[35] T. W. Cain , C. F. Glover , J. S. Laird , N. Birbilis , J. R. Scully , Corrosion 2021, 77, 115.

[36] M. Esmaily , J. Svensson , S. Fajardo , N. Birbilis , G. Frankel , S. Virtanen , R. Arrabal , S. Thomas , L. Johansson , Prog. Mater. Sci. 2017, 89, 92.

[37] A. D. Südholz , N. T. Kirkland , R. G. Buchheit , N. Birbilis , Electrochem. Solid‐State Lett. 2011, 14, C5.

[38] S. Fajardo , G. Frankel , Electrochim. Acta 2015, 165, 255.

[39] M. Grimm , A. Lohmüller , R. F. Singer , S. Virtanen , Corros. Sci. 2019, 155, 195.

[40] P. Gore , T. W. Cain , J. Laird , J. R. Scully , N. Birbilis , V. S. Raja , Corros. Sci. 2019, 151, 206.

[41] T. Cain , S. B. Madden , N. Birbilis , J. R. Scully , J. Electrochem. Soc. 2015, 162, C228.

[42] N. Birbilis , R. G. Buchheit , J. Electrochem. Soc. 2015, 152, B140.

[43] M. Esmaily , J. E. Svensson , S. Fajardo , N. Birbilis , G. S. Frankel , S. Virtanen , R. Arrabal , S. Thomas , L. G. Johansson , Prog. Mater. Sci. 2017, 89, 92.

[44] T. Cain , L. G. Bland , N. Birbilis , J. R. Scully , Corrosion 2014, 70, 1043.

[45] N. Birbilis , A. King , S. Thomas , G. Frankel , J. Scully , Electrochim. Acta 2014, 132, 277.

[46] T. Cain , C. Glover , J. Scully , Electrochim. Acta 2019, 297, 564.

[47] T. W. Cain , C. F. Glover , J. R. Scully , Corrosion 2023, 79, 1360.

[48] G. Song , A. Atrens , Adv. Eng. Mater. 1999, 1, 11.

[49] G. Song , A. Atrens , Adv. Engergy Mater. 2003, 5, 837.

[50] G. Song , A. Atrens , F. Cao , Z. Shi , P. K. Bowen , J. Magnesium Alloys 2013, 1, 177.

[51] R. L. Liu , M. F. Hurley , A. Kvryan , G. Williams , J. R. Scully , N. Birbilis , Sci. Rep. 2016, 6, 28747. 27350286 10.1038/srep28747 PMC4923887

[52] S. Fajardo , J. Bosch , G. Frankel , Corros. Sci. 2019, 146, 163.

[53] T. W. Cain , I. Gonzalez‐Afanador , N. Birbilis , J. R. Scully , J. Electrochem. Soc. 2017, 164, C300.

[54] M. Esmaily , J. Svensson , S. Fajardo , N. Birbilis , G. Frankel , S. Virtanen , R. Arrabal , S. Thomas , L. G. Johansson , Prog. Mater. Sci. 2017, 89, 92.

[55] A. Bahmani , S. Arthanari , S. K. Seon , J. Magnesium Alloys 2020, 8, 134.

[56] K. Wang , C. Li , Y. Li , J. Lu , Y. Wang , X. Luo , J. Magnesium Alloys 2021, 9, 866.

[57] Z. Shen , M. Zhao , D. Bian , D. Shen , X. Zhou , J. Liu , H. Guo , Y. Zheng , J. Mater. Sci. Technol. 2019, 35, 1393.

[58] C. Punckt , S. Bölscher , H. H. Rotermund , A. S. Mikhailov , L. Organ , N. Budiansky , J. R. Scully , J. L. Hudson , Science 2004, 305, 1133. 15326349 10.1126/science.1101358

[59] S. Jain , N. D. Budiansky , J. L. Hudson , J. R. Scully , Corros. Sci. 2010, 52, 873.

[60] S. Jain , J. L. Hudson , J. R. Scully , Electrochim. Acta 2013, 108, 253.

[61] G. Williams , N. Birbilis , H. N. McMurray , Electrochem. Commun. 2013, 36, 1.

[62] G. S. Frankel , A. Samaniego , N. Birbilis , Corros. Sci. 2013, 70, 104.

[63] F. Xia , J. Liu , H. Nie , Y. Fu , L. Wan , X. Kong , IEEE Trans. Emerging Top. Comput. Intell. 2020, 4, 95.

[64] N. DeTal , A. Kaboudian , F. H. Fenton , Proc. Natl. Acad. Sci. USA 2022, 119, e2117568119. 35679346 10.1073/pnas.2117568119 PMC9214532

[65] K. Gallagher , M. A. Strobl , D. S. Park , F. C. Spoendlin , R. A. Gatenby , P. K. Maini , A. R. Anderson , Cancer Res. 2024, 84, 1929. 38569183 10.1158/0008-5472.CAN-23-2040 PMC11148552

[66] J A. Roberts , L L. Gollo , R G. Abeysuriya , G. Roberts , P B. Mitchell , M W. Woolrich , M. Breakspear , Nat. Commun. 2019, 10, 1056. 30837462 10.1038/s41467-019-08999-0 PMC6401142

[67] S. Lebouil , O. Gharbi , P. Volovitch , K. Ogle , Corrosion 2015, 71, 234.

[68] D. Barkley , Phys. D 1991, 49, 61.

[69] D. Barkley , Phys. Rev. Lett. 1994, 72, 164. 10055592 10.1103/PhysRevLett.72.164

[70] L. Rossrucker , A. Samaniego , J.‐P. Grote , A. M. Mingers , C. A. Laska , N. Birbilis , G. S. Frankel , K. J. J. Mayrhofer , J. Electrochem. Soc. 2015, 162, C333.

[71] G. Cheng , H. Hu , W. E. Frazier , C. A. Lavender , V. V. Joshi , Mater. Sci. Eng. A 2018, 736, 41.

[72] R. Akid , M. Garma , Electrochim. Acta 2004, 49, 2871.

