# Supplementary material for: Morphogenic Modeling of Corrosion Reveals Complex Effects of Intermetallic Particles
Source: Adv Sci (Weinh). 2024 Aug 19;11(39):2404986. doi: 10.1002/advs.202404986 (PMC11496997; doi:10.1002/advs.202404986)
Supplement: Supplementary file 1 — Supporting Information [file ADVS-11-2404986-s001.pdf]

ADVANCED SCIENCE
Open Access

Supporting Information

for *Adv. Sci.*, DOI 10.1002/advs.202404986

Morphogenic Modeling of Corrosion Reveals Complex Effects of Intermetallic Particles

Bruno C. Batista, Elena Romanovskaia, Valentin Romanovski, Michael Emmanuel, James T. Burns, Ji Ma, Istvan Z. Kiss, John R. Scully* and Oliver Steinbock**

SUPPORTING INFORMATION

Morphogenic modeling of corrosion reveals complex effects of intermetallic particles

Bruno C. Batista[1], Elena Romanovskaia[2,4], Valentin Romanovski[2,4], Michael Emmanuel[3], James T. Burns[2], Ji Ma[2], Istvan Z. Kiss[3*], John R. Scully[2*], and Oliver Steinbock[1*]

1) Florida State University, Department of Chemistry and Biochemistry, Tallahassee, FL 32306, USA
2) University of Virginia, Center for Electrochemical Science and Engineering, Department of Materials Science and Engineering, Charlottesville, VA 22904, USA
3) Saint Louis University, Department of Chemistry, 3501 Laclede Ave., St. Louis, MO 63103, USA
4) These authors contributed equally: Elena Romanovskaia, Valentin Romanovski.

* Corresponding authors: Oliver Steinbock: osteinbock@fsu.edu, Istvan Z. Kiss: istvan.kiss@slu.edu, John R. Scully: jrs8d@virginia.edu

Content: Tables S1, S2; Figures S1-S13; Movie S1; additional text

Materials and Supplies

The Mg alloy AZ31B-H24 was obtained from Metalmart International, Inc. (Mill: Luxfer/Magnesium Elektron). The alloy composition is given in Table S1. The solutions in our corrosion experiments were 0.6 M NaCl. All solutions were prepared using reagent-grade NaCl and deionized water.

Table S1. Composition (wt%) of the material used in this study as provided by the supplier.

Alloy	UNS#	Mg	Al	Zn	Mn	Si	Ni	Fe	Cu
AZ31B-H24	M11311	Balance	3.12	1.09	0.42	0.015	0.0012	0.0029	0.001

Samples Preparation for Analysis

The samples were cut from a bulk piece of AZ31B. The ST surface was selected for analysis. All samples were polished using silicon carbide paper with grit down to 1200. The polished samples were sonicated in deionized water (18.2 MΩ·cm) and isopropanol for 3 min before experiments. For SVET analysis samples were molded into epoxy to fit the SVET electrochemical cell.

IMP Characterization

The IMPs' number density, size distribution, and compositional distribution, as well as their corrosion initiation properties, were analyzed using a dual-beam scanning electron microscope (SEM) Helios UC G4 (Thermo Fisher Scientific, USA) and Themis 60-300 kV Transmission Electron Microscope (TEM) (Thermo Fisher Scientific, USA). Sample cross-sections and thin slices for detailed TEM analyses at nanoscale resolution were prepared with a Focused Ion Beam (FIB) system on the Helios UC G4. The Helios offers Energy Dispersive X-ray Spectroscopy (EDS) for compositional analysis. The Themis system from Thermo Fisher Scientific allows for imaging of materials with atomic resolution in both the Transmission Electron Microscopy (TEM) and the probe-corrected Scanning TEM (STEM) modes. Compositional analyses and mapping were obtained using Energy Dispersive X-Ray Spectroscopy (EDS) and Electron Energy-Loss Spectroscopy (EELS). As a minimum, 300 IMPs (up to 800) were analyzed to obtain insights about compositional distribution.

Electrochemical measurements

All full immersion tests were conducted in 0.6 M NaCl over a 1 cm^2 surface area. Open circuit potential experiments (OCP) were carried out on a Gamry 620 potentiostat with computer interface software. A three-electrode cell with a saturated calomel electrode (SCE) as reference (e.g. +.241 V vs a standard hydrogen electrode) and Pt mesh as a counter electrode were used for all electrochemical tests. Open circuit experiments were performed for up to 30 days for AZ31B, and for 24 h for IMPs with a 1 s step.

Table S2. Composition and relative abundance by the count of intermetallic particles (IMPs) in AZ31B. Measurements are performed for IMPs with diameters above 10 µm.

**IMP Type**	**Fraction %**	**Open Circuit Potential V_{sce} (V)**
AlMnFe	47.9	-1.06±0.10
AlMn	38.9	-1.13±0.27
Si	5.6	-0.45±0.00
MgMnFe	0.7	unavailable
AlMn(Fe)Si	2.1	-0.85±0.03
Mn	2.1	-1.32±0.00
Fe	2.8	-0.65± 0.1

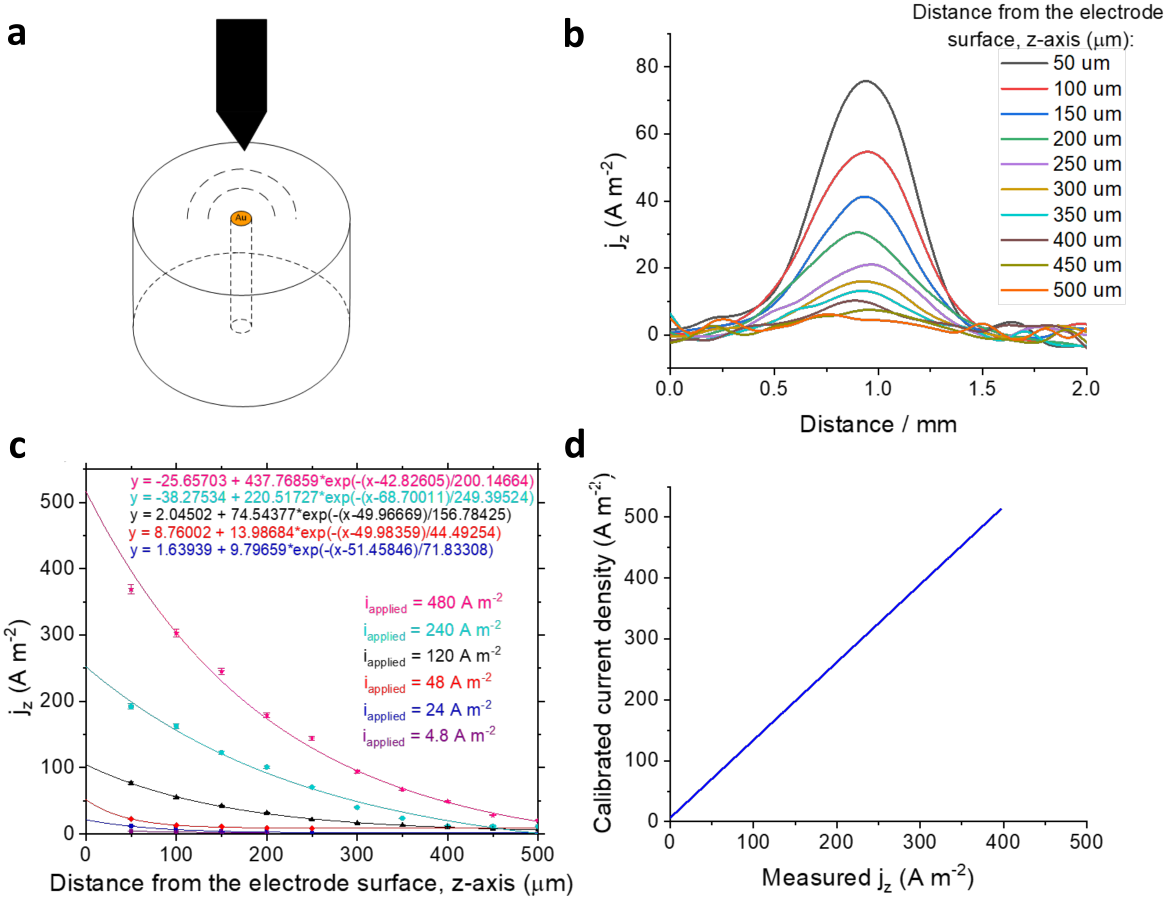

Figure S1. Calibration of the SVET data by the distance of the probe above the sample using a gold point current source: (a) schematic of the Au point current source and SVET probe position; (b) the distribution of the SVET-derived current density depending on the *z*-distance of the probe above the Au point current source surface at an applied current density of 120 A/m^2; (c) the distribution of the SVET-derived peak current densities depending on the *z*-distance of the probe above the Au point current source surface at different applied current densities; (d) calibration line for the SVET-derived current densities at a *z*-distance of 50 µm.

Profilometry Measurements

The corroded surface morphology was investigated by 3D microscopy (Hirox, Hirox USA Inc.) under a magnification of ×150 with a detection limit of 1 μm^2. Hirox RH-8800 Light Microscope provides nondestructive digital imaging, videography, and measurement of various material surfaces under magnification up to ×5000. Stage tilt (45 degrees) for inspection of surface roughness, 360 degrees of rotation for optimal understanding of object shape and 3-D modeling. The analyzed surface area was 1 cm^2 for each sample. Obtained raw data was processed using in-house MATLAB R2020a scripts.

Optical Detection of Corrosion Waves

While most experimental data on the propagation of corrosion waves are obtained by SVET, the corrosion waves on salt-solution-covered AZ31B samples can also be discerned by the unaided eye. Figure S2 shows a typical image sequence of such a traveling wave recorded by a digital microscope camera (MS1; Hayve, China; resolution: 1920×1080 pixels). Notice that, as expected, the wave anodic activity is coupled to the cathodic production of hydrogen that nucleates streams of very small bubbles along the moving corrosion front (see also Movie S1). Corrosion waves appear as dark regions which grow and spread with time.

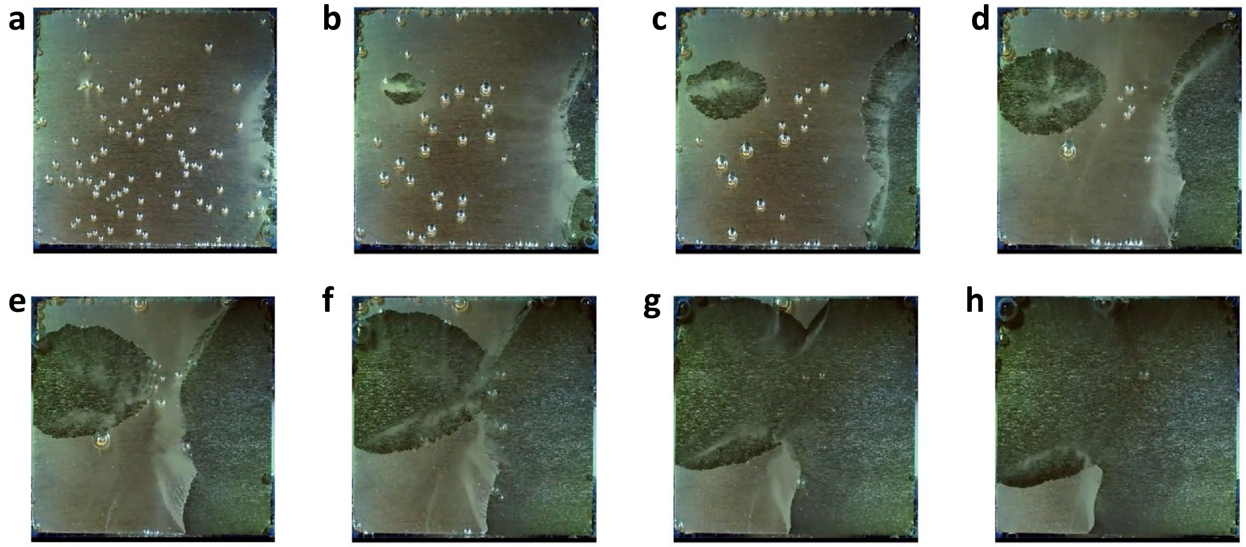

Figure S2. Sequence of optical images of the AZ31B surface showing the propagation of a corrosion wave. While not used for analyses, these data complement our SVET results (see Fig. 1b,c). Time between frames: 1440 s. Field of view: 2×2 cm^2. See also Movie S1.

Intermetallic Particles

It is well known that intermetallic particles (IMPs) can play a significant role in the corrosion behavior of alloys. These particles often have different electrode potentials and coupled corrosion potentials (i.e., open circuit potentials) than the matrix based on their differing chemical contents as well as structure

[ref. 42], creating microgalvanic couples that can initiate corrosion. This localized corrosion produces Mg(II) species and this is a trigger drawing in more Cl^- to maintain electroneutrality. Corrosion is thus auto-catalytic and can then propagate by speading the active corrosion front away from the IMP over time and its best visual observation is on Mg and Mg-alloys [S1] (see supplementary informational video file). This corrosion wave in Figure S2 fully corresponds to the wave seen via SVET analysis [S2] (Figure S3). Examination of IMPs inside the initiation sites on AZ31B alloy in 0.6 M NaCl (Figure S1a) showed that corrosion was initiated around the AlMnSi (Spot 1) and AlMn (Spot 2) particles. The electrode potentials of similar IMPs reported in the literature such as $Al_{12}Mn_3Si$ and Al_6Mn are –0.858 V and –0.913 V vs. a saturated calomel electrode which provides a thermodynamic and kinetic driving force for waves [S3]. Therefore, IMPs with more positive electrode potentials initiate the corrosion and the resulting corrosion waves observed on Mg and Mg alloys. These corrosion waves correspond to those detected by SVET (Figure S3b,c). Note that not all IMPs lead to sustained waves (single bubbles). This is due to the large range of IMP electrochemical properties reported in Table S2. Some IMPs produce a galvanic cell (AlMn and AlMnSi as shown above) with a higher current density and these, in turn, have a higher chance of spreading, while other IMPs in Table 2 do not.

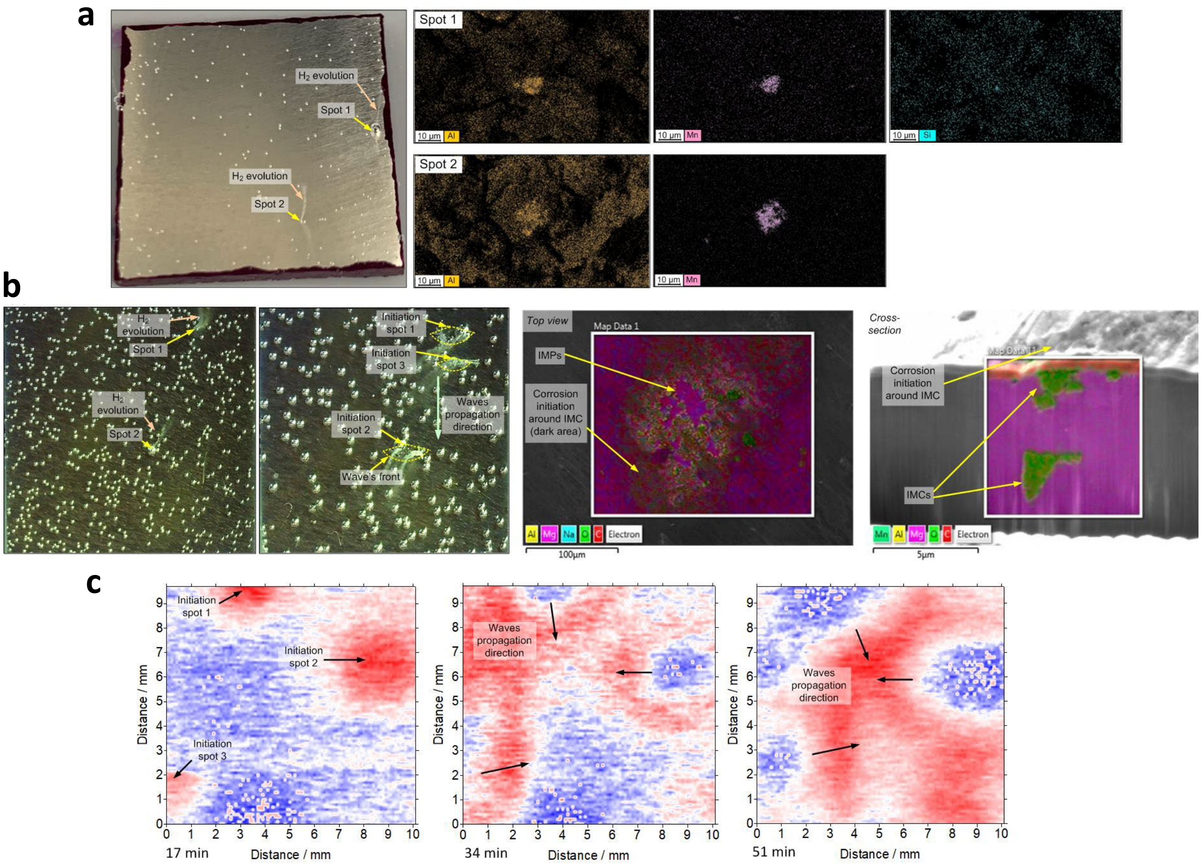

Figure S3. Example of IMPs that initiate (a) corrosion and (b,c) wave propagation over the AZ31B sample surface. The data in (a,b) are obtained by optical microscopy and EDS, while the maps in (c) are measured by SVET. Results are from three separate experiments.

Processing of the SEM Data

We analyze six representative electron backscatter SEM images of our AZ31B samples. Their spatial resolution is 5.175 pixel/µm and each image has a pixel size of 3840×2460. Using an in-house MATLAB script, we apply an intensity threshold to the images to obtain binary versions in which 0 and 1 denote the background and the intermetallic particles, respectively. Each binary image is then processed further using the watershed algorithm [S3] to conservatively split bright areas that correspond to separate IMPs that are detected as one connected area. The latter step increases the number of detected particles by about 6%. An example illustrating this process is shown in Figure S4.

Using these processed images, we perform a blob analysis (MATLAB command bwlabel), which identifies and labels connected components (blobs). Their areas A are then converted to equivalent radii $r_{eq} = \sqrt{A/\pi}$ and fit to inverse Gaussian distributions

$$f(r|\mu,\lambda) = \sqrt{\frac{\lambda}{2\pi r^3}} \exp\left(-\frac{\lambda(x-\mu)^2}{2\mu^2 r}\right).$$

The result of this analysis is shown in Fig. 1a (main paper) yielding a mean μ = 0.909 µm and a shape parameter λ = 0.385 µm.

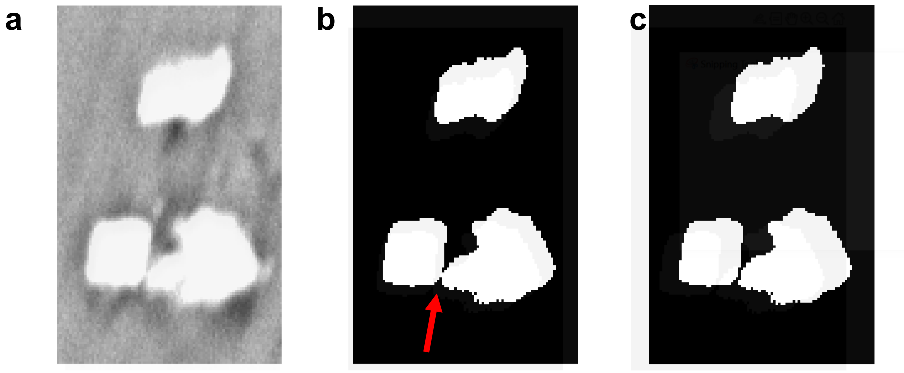

Figure S4. Illustration of the watershed method. (a) Small area of the original SEM (contrast linearly increased without information loss). (b) The same area after application of the intensity threshold. The two lower particles are connected (red arrow). (c) The same binary image after processing with the watershed algorithm. The two lower particles are now unconnected. Image size: 17.4×27.8 µm^2.

Comparison to a Simple Random Process

Both our experimental and modeling results (Matlab R2020a) suggest that the surface variance increases linearly over time (Fig. 1f and Figure S5). Notice that the variance of profile $z(x)$ is the square of the surface roughness (if the latter is defined as the standard deviation of z).

This linear dependence is also found in one of the simplest stochastic models of surface erosion. Figure S5a shows a simple calculation in which the initial constant profile $z(x)$ is represented by $N = 2000$ discrete sites. With each time step, one of these sites is randomly selected and its value is decreased by one. Over time, this process yields white-noise profiles at average values that change at a rate of $dz/dt = -1/N$ per time step. Accordingly, after $1.5 \cdot 10^5$ time steps, the corresponding purple curve is at an average value of $1.5 \cdot 10^5/2000 = 75$.

As expected, for this elementary statistical process, the corresponding variance is proportional to time (Figure S5b) and hence recovers, despite its extreme simplicity, these aspects of our experimental and modeling results. Notice that the white-noise characteristics of the results, however, do neither agree with experimental nor modeling results.

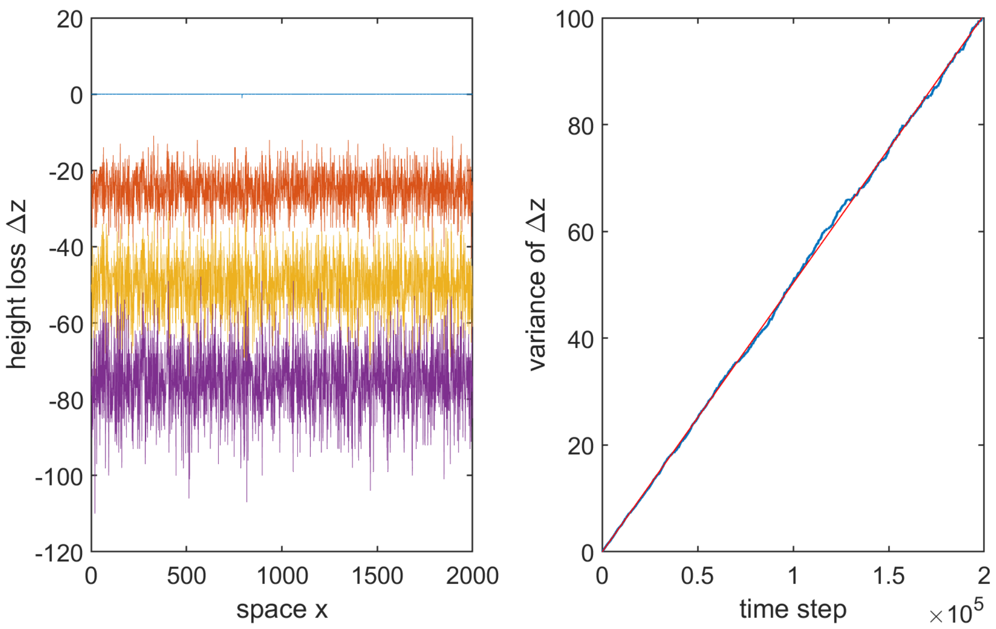

Figure S5. An elementary stochastic model of corrosion processes. For each time step, one of the N = 2000 sites is randomly selected to change in height by $\Delta z = -1$. (a) Representative surface profiles after 0 (blue), $5 \cdot 10^4$ (orange), $1 \cdot 10^5$ (gold), and $1.5 \cdot 10^5$ time steps (purple). (b) The variance of the $z(x)$ profile as a function of time (blue) obeys a proportional dependence (best linear fit is shown in red).

Simulated Surface Waves

All of our simulations use the following model parameters: ε = 0.02, a = 0.44, and b = 0.045. These parameters set the time-scale difference (ε) in the dynamics of the u and v variables and the system's excitation threshold (b/a). The chosen values define a system that allows the steady propagation of excitation waves while excluding the possibility of compact spiral waves which are a frequent solution in this class of nonlinear models [S4] but have not been observed in experiments with corroding AZ31B surfaces. This absence of tightly wound spiral waves is related to the dynamics of open wave ends which can form when waves encounter a corrosion-protected or refractory area. For spiral waves, the wave ends tangentially expand but—due to curvature—move slower than an undisturbed planar front. Accordingly, the wave segments near the ends fall behind and curl into a pair of spirals. In our system, however, the wave ends expand so slowly that the trajectory of the spiral tip would form an orbit much larger than the system size. This behavior qualitatively matches our experimental observations for corrosion waves on AZ31B surfaces exposed to NaCl solutions and agrees with the absence of experimental reports of spiral waves on the corroding alloy. Notice that the wave ends can also laterally shrink, constituting spiral-free subexcitable behavior. This rather similar type of dynamics was not studied but can be selected by a small reduction of the chosen a value and/or a slight increase in b.

The model parameters k and ζ in the z-equation are varied between different simulations but affect neither the u, v dynamics nor the shape of the wave pulses in IMP-free settings. Figure S6 shows the evolution of the model variable u in a simulation for such an IMP-free surface. We emphasize that these two-dimensional model calculations describe the solution-exposed 2D surface of the alloy, while our main study focuses on 2D lamellae of the material with a solution-exposed 1D interface (see Fig. 2a). For these 1D interface dynamics, the wave tip behavior in 2D has no direct importance but justifies our selection of parameters.

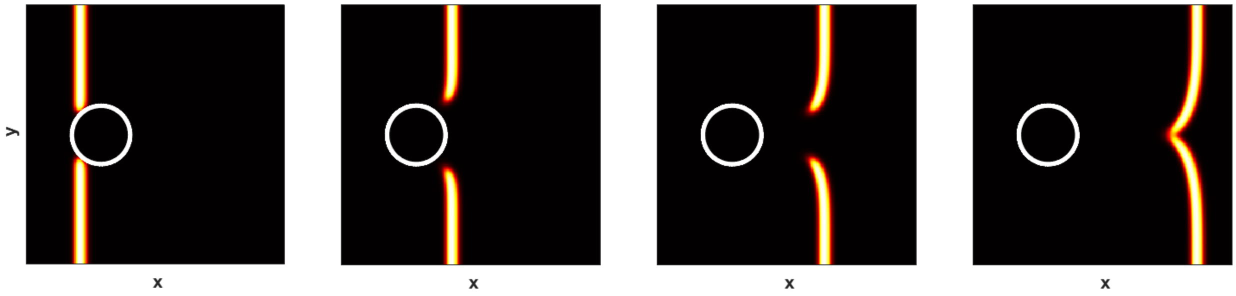

Figure S6. Two-dimensional simulation of the unscaled, core model without material loss in an IMP-free system. The four frames show an image sequence illustrating the collision of a planar wave (orange bands) with an inert circular domain (white ring) and the dynamics of the open wave ends in the wake of the inert obstacle. The very slow expansion of the rightward moving wave ends is similar to the wave dynamics of open wave ends observed in AZ31B experiments. Model parameters: ε = 0.02, a = 0.44, b = 0.045, and k = ζ = 0. The inert disk is created by setting v = 0. The simulation domain consists of 250 $\times$ 250 grid points spaced at Δx = 0.15 space units. Integration time step: Δt = 0.0025 time units.

Model Calibration

Using a numerical lattice with a constant grid spacing of Δx = 1 µm and a time step of Δt = $1\cdot10^{-6}$ time units, we calibrate our model according to experimental measurements obtained at 0.6 M NaCl. These experiments yield a wave width of 1 mm and a wave speed of 120 µm/min. The calibration first yields 1 µm = 0.002 space units and then, in a second step, $1\cdot10^{-6}$ time units = $1.4\cdot10^{-5}$ min. The calibrated wave is illustrated in Figure S7a,b which shows a rightwards moving pulse in terms of the variables *u* and *v* and its time-space plot, respectively. The wave width is identified as the width of the fully developed *u* pulse at half-height and the wave speed is obtained from the front speed.

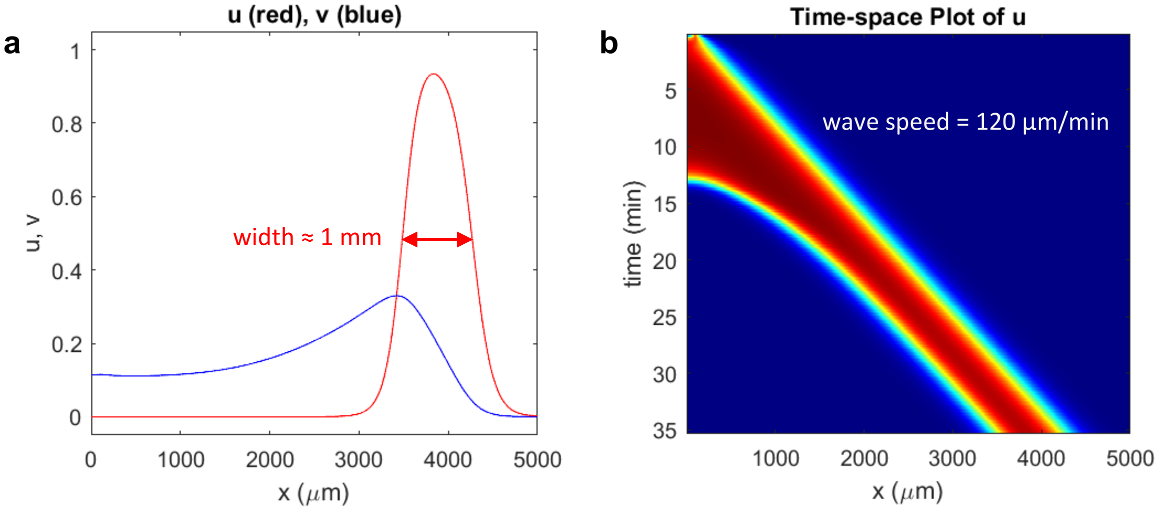

Figure S7. Calibrated wave pulse. (a) Profiles of the model variables *u* and *v*. The wave width is only slightly below the target of 1 mm. (b) Time-space plot of *u* with red and blue areas indicating high and low *u* values, respectively. The front speed equals the experimental value of 120 µm/min.

Attempted Construction of Surface Profiles from IMP Maps Alone

We attempted to construct surface profiles $z(x)$ from our simulated IMP maps without the use of nonlinear waves and similar response modes of our full model. For this, we interpret the IMP map as a binary array $I(x,z)$ where I = 0, 1 implies the absence or presence of an IMP at the alloy site (x,z), respectively. Like in our regular simulations, these IMP maps are based on the experimentally determined size distribution (Fig. 1a) and number density of 880 IMPs/mm^2. The spatial resolution is 1 µm and the map covers 10×0.28 mm^2, which (at the average corrosion rate of 0.02 µm/min) would corrode in about 14 days. As in most simulations except for Fig. 5, the IMPs are circular and their positions are assumed to be uncorrelated.

We then apply a Gaussian filter G_σ to $I(x,z)$ to approximate the spread-out effects of the particles on the corroding surface. The standard deviation or width of the Gaussian filter is denoted as σ and varied between 0 (no filter) and 1 mm. To minimize artifacts near the system boundaries (x = 0 and 10 mm), we

pad the data with mirror images of the original IMP map. Next, the smoothed IMP map $G_\sigma(I(x,z))$ is collapsed to a one-dimensional vector $z_G(x)$ by summation over all z according to $z_G(x) = \Sigma\, G_\sigma(I(x,z))$. This step estimates the cumulative effect of IMP-dependent corrosion on the evolving alloy-solution interface. Notice that this approach does not consider the roughness of the interface but rather assumes that the height changes along the corroded surface are small compared to the average depth of lost material. Moreover, we did not attempt to scale the resulting z_G values to match the amplitude of the measured curves.

Figure S8 shows representative results obtained by this approach. For panel (a), no Gaussian filter is applied and the surface is reminiscent of uncorrelated white noise which agrees with neither the experimental nor the reaction-diffusion model outcomes. Panels (b-d) show the computed surface profiles for filter widths of σ = 87, 250, and 533 µm, respectively. Especially for the data in (b), we find some agreement with the corresponding surface profile obtained by numerical simulation of our reaction-diffusion model (eqs. 1 in main paper), which is shown in (e, f). Notice that the latter panels are identical and duplicated only to allow an easier visual comparison within each figure column.

To obtain a more objective comparison between the two methods, we compute the respective correlation coefficients for different filter widths σ (Figure S8g). The maximal correlation is 0.56 (marked by a blue star) and found for σ = 87 µm, the value used for panel (b). This correlation coefficient reaffirms our qualitative impression of a certain level of agreement but also shows the limitation of this simple (yet very rapid) computational approach. We also tested Lorentzian kernels and obtained similar results. In summary, we conclude that the effects of IMPs on the corrosion dynamics are roughly determined by their locations, but knowledge of the surface's dynamic state during the entire course of the corrosion process is needed to evaluate how consequential individual IMPs are. Our Gaussian filter approach might prove useful for certain tasks, but—due to its lack of a physical foundation—it seems unlikely that it will guide the development of mitigation strategies. However, such guidance can be obtained from our nonlinear reaction-diffusion model.

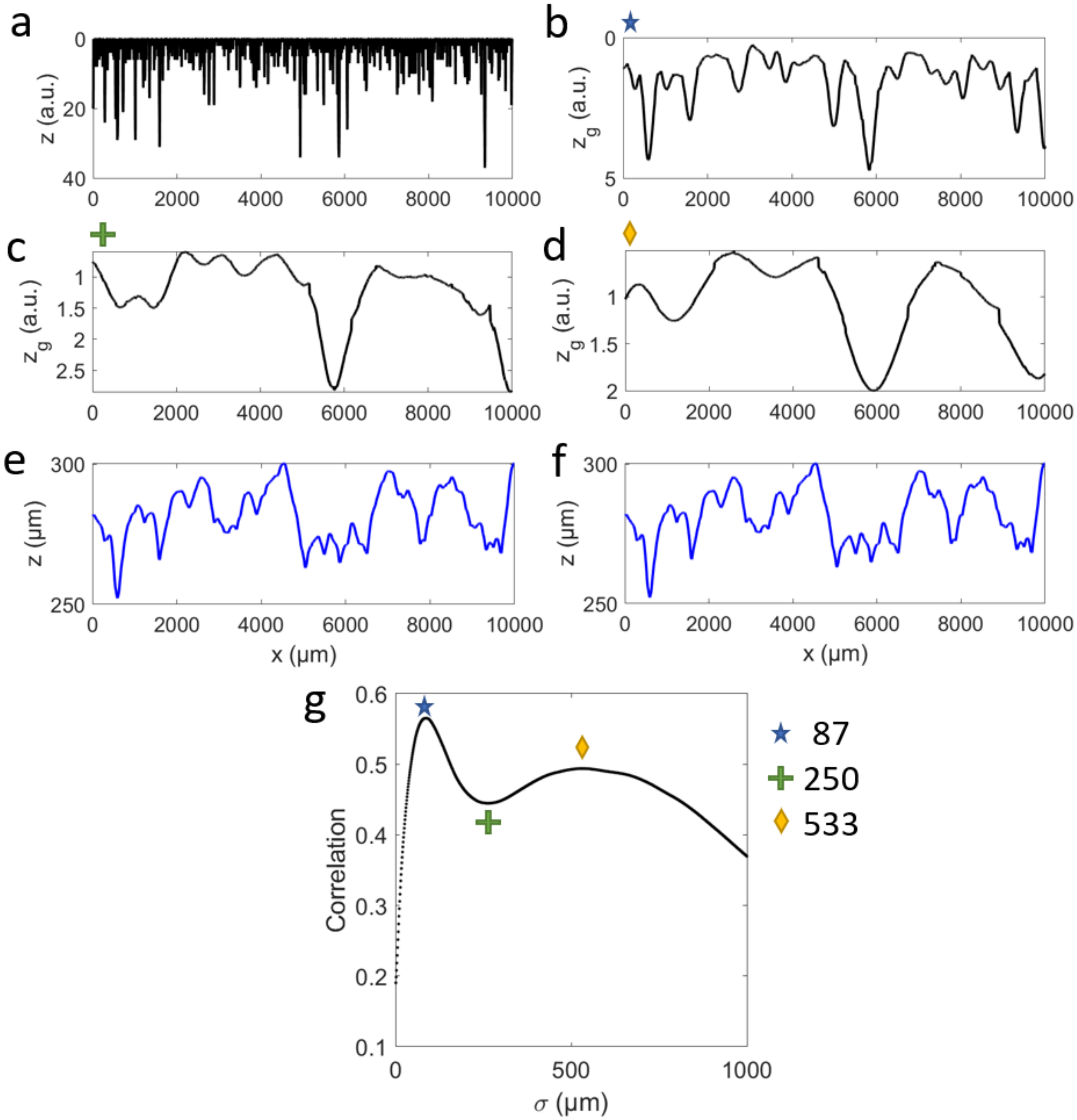

Figure S8. Damage profiles generated solely from (a) collapsed IMP maps and (b-d) additional Gaussian filters with standard deviations of σ = 87, 250, and 533 µm, respectively. The profiles only vaguely resemble the experimental data in Fig. 1 and—despite being derived from the same IMP map—also differ from the numerical results (e,f) obtained from our nonlinear reaction-diffusion model (eqs. 1 in main paper). However, some similarities exist, especially between panels (b) and (f). (g) Correlation coefficient for the profiles obtained by the Gaussian-filter method and our full-model data for different filter widths σ. Colored markers indicate the specific σ values used in panels (b-d). Notice that panels (e,f) are identical.

Corrosion Dynamics without Background Corrosion

In systems without any background corrosion ($\zeta = 0$), the surface loss tends to stall as it cannot reach the next lowest IMP that would reignite the corrosion process. Very large k values, however, cause the dynamic surface to leapfrog from IMP to IMP and sustain the corrosion process for long periods. This corrosion regime is not applicable to AZ31B but could possibly exist in other alloys.

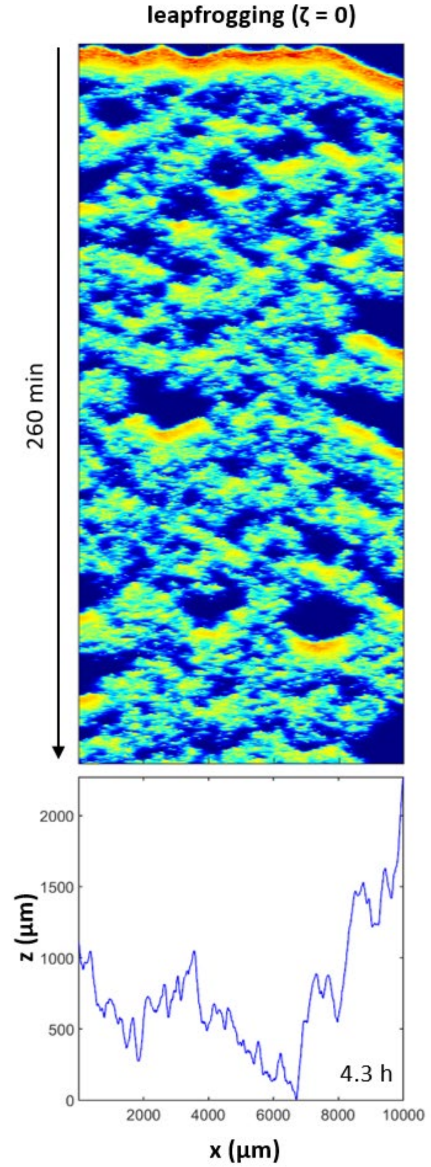

Figure S9. Leapfrogging corrosion dynamics. The top panel is a time-space plot showing the temporal evolution of $u(x)$ with time increasing in the downward direction. The lower panel shows the surface profile after 4.3 h. This corrosion regime complements the scenarios shown in Fig. 3. Parameters: $k = 150$ μm/min, $\zeta = 0$, and $u_{IMP} = 0.5$.

Simulations of Several Weeks of Corrosion Activity and Long-term Evolution of the Surface Variance

Our model allows the fast simulation of AZ31B lamellae spanning 1 cm (or more) while realistically resolving the micron-scale structure of the involved IMPs in the alloy. For example, the time required to compute 82 days of corrosion dynamics (Figure S10) is about 50 hours on a personal computer (processor: Intel Core i9-12900, 2400 MHz; 64 GB RAM), which is equivalent to a time compression factor of approximately 40. Despite large fluctuations, such long-term simulations show that the variance of the surface profile (i.e. the square of the surface roughness) increases linearly over time (Figure S10b).

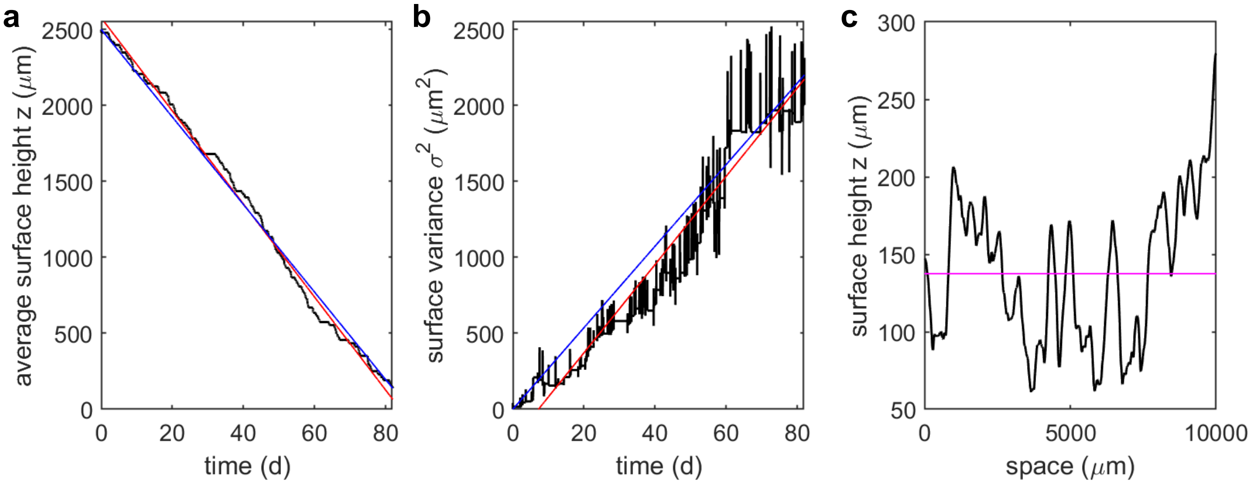

Figure S10. Simulation of 82 days of corrosion activity. (a) Average surface height as a function of time (black). The red and blue curves are the linear regression line and the line connecting the first and last point, respectively. The corresponding corrosion rate (negative slope of red curve) is 0.0213 µm/min. (b) Variance of the surface height $z(x)$ as a function of time (black). Notice that the variance is the square of the surface roughness, if defined as the standard deviation σ. The red and blue curves are the linear regression line and the line connecting the first and last point, respectively. (c) Surface profile after 82 days (black) with the magenta line indicating the average. The initial height profile was z = 2500 µm. Model parameters: $\zeta = 2.32 \cdot 10^{-4}$ µm/min, k = 3 µm/min, and u_{IMP} = 0.6.

Computer-generated IMP Distributions

As expected from earlier studies, our SEM images show random distributions of IMPs. The shape and size of the IMPs also vary. In addition, IMPs can be arranged along streaks or lines that were created in the breakup of large particles during the rolling of the alloy. Accordingly, the locations of the IMPs in AZ31B show small deviations from a uniform random distribution as well as some anisotropy.

a b

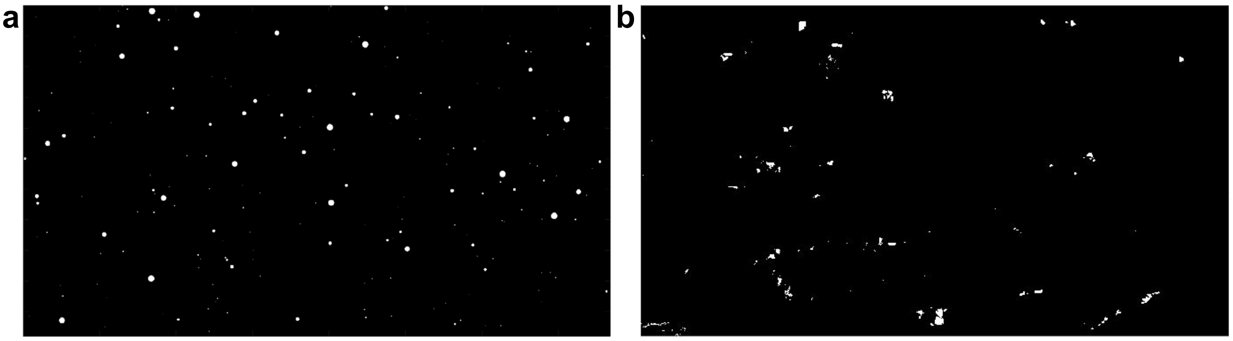

Figure S11. (a) Simulated IMP map without spatial correlation and (b) processed SEM image. Field of view: 742×417 μm^2. Number of IMPs: (a) 272 (potential overlap of particles is ignored) and (b) 296.

We employ two different methods for generating IMP maps for our corrosion simulations. These maps are binary arrays with 0 and 1 denoting the IMP-free alloy and the IMP, respectively. In both methods, the IMPs are circular disks with a radius distribution that is generated as random numbers obeying the experimentally determined inverse Gaussian distribution. We control these random radii by selecting seeds that allow the repeated use of the same IMP map as well as controlled comparisons between a few different maps. Notice that IMPs with sizes below the spatial resolution of the simulation are not considered. In addition, very large IMPs (r > 20 µm) are not considered, although the mathematical distribution can generate such random numbers with a low probability. The rationale behind this truncation is that such particles are unlikely in experiments and would generate anomalous examples.

The difference between methods is the placement of the IMP centers on the map grid. For the corrosion simulations in Figs. 3-5, the centroids are selected as uniformly distributed random numbers. Accordingly, there is no spatial correlation and no large-scale anisotropy. For the corrosion simulations in Figure S13, however, we use IMP maps that capture the qualitative features of the IMP stringers. The method for generating these maps is explained in the following.

We first estimate the following parameters:

- Maximal number of IMPs per stringer: AN = 30
- Maximal length of a stringer in micrometers: AL = 250
- Maximal tilt angle of the stringer axis with respect to the L direction: AP = 2 degrees
- Parameter controlling the width of the stringer: AS = 5.8
- Target number of IMPs NS for the selected area based on the measured number density of 880 IMPs/mm^2.

By generating uniformly distributed random numbers ξ^*, we then iteratively create stringers with $\xi^*\times$AN IMPs distributed along a target line of length $\xi^*\times$AL where the target line has a small tilt of $(2\xi^*-1)\times$AP. In addition, each IMP centroid is moved off the target line in the normal direction by a small shift equaling a random number from a Gaussian distribution. The standard deviation of this distribution is (AS$\times\xi^*$+10) µm.

Evaluation and Use of the Computer-generated "Stringer" Maps

Figure S12 shows representative examples of processed SEM images (left column) and computer-generated IMP maps (right column). Visual comparison of the images indicates strong qualitative similarities. Notice the presence of relatively large IMP-free regions as well as the local clustering and streaking of particles.

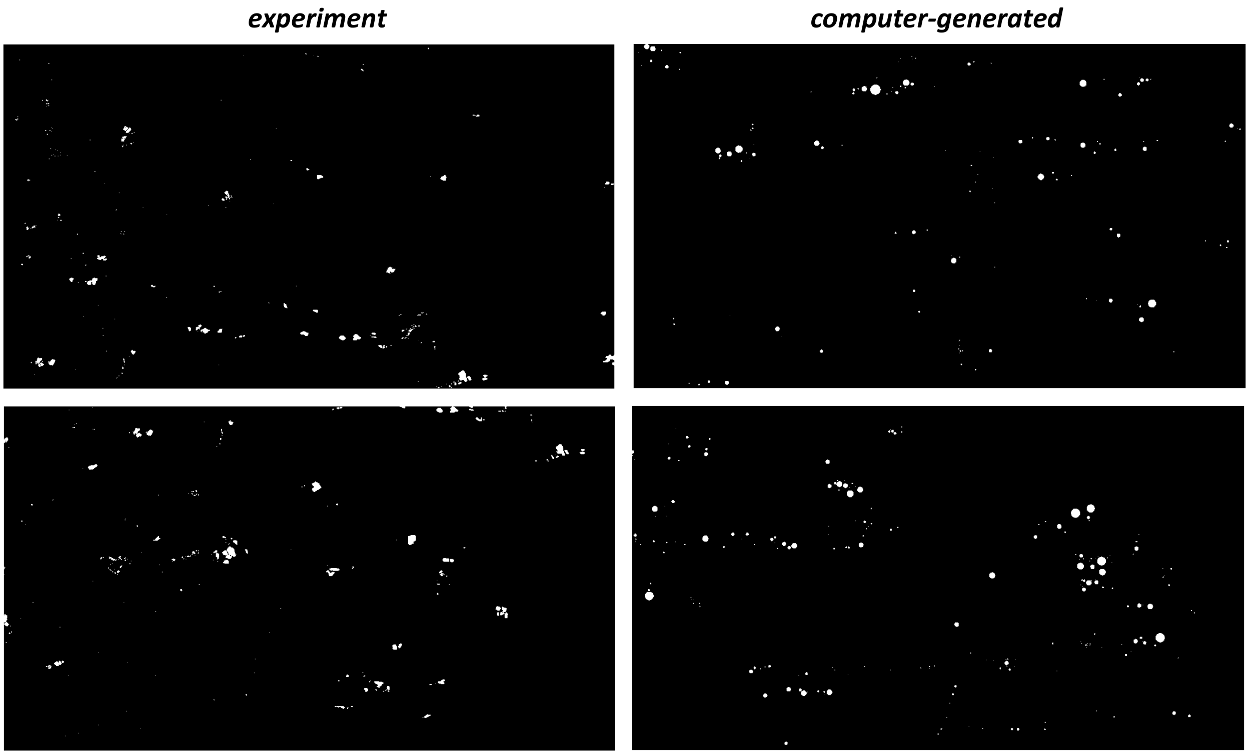

Figure S12. Comparison of processed SEM images (left column) and simulated IMP patterns (right column). All image sizes: 742×417 µm^2.

Applying stringer maps to our nonlinear reaction-diffusion model (eqs. 1) produces results that closely match those from original maps with uncorrelated particle positions (Figure S13). To compare the two simulation types (or IMP maps), we performed two-sample t-tests on both dz/dt and dσ^2/dt coordinates, assuming normal distribution and equal variances. These tests, standard for comparing means of two groups, show no significant differences between Class A and Class B in both coordinates, with p-values of 0.755 and 0.274, respectively. The hypothesis test results, being 0 for both coordinates, suggest insufficient evidence at the 5% significance level to reject the null hypothesis of no significant difference in corrosion behaviors between the two IMP map types.

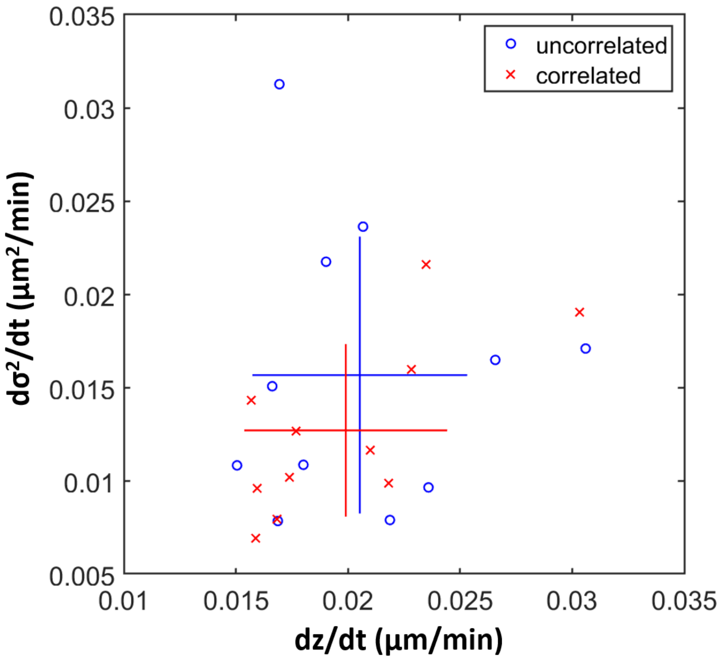

Figure S13. Comparison of the simulated average material loss rate (dz/dt) and the increase in surface variance (dσ^2/dt) for IMP maps with particles placed according to uniform random numbers (blue) and particles showing stringer-like features (red). The individual markers indicate the results obtained from simulations with IMP maps that differ only in the seed of the random number generator (i.e. different maps that obey the same statistical parameters). The central points of the plus-shaped error bars and the error bars themselves represent the corresponding averages and standard deviations. The very small differences between the average points fall within one standard deviation. This finding suggests that stringers, when compared to uncorrelated IMP placements, do not affect the average corrosion rate or surface roughening rate strongly. Model parameters: $\zeta = 2.32 \cdot 10^{-4}$ µm/min, k = 3 µm/min, and u_{IMP} = 0.6.

Movie S1

Time-lapse movie showing corrosion waves and gas bubble formation on an NaCl(aq)-exposed AZ31B surface. Field of view: 2×2 cm^2. Total time shown: 4 h.

References Used in SI

[S1] M. Esmaily et al., *Progr. Mater. Sci.* **2017**, *89*, 92-193.

[S2] T. W. Cain et al., *Corrosion* **2023**, *79*, 1360-1378.

[S3] N. Birbilis and R. G. Buchheit, *J. Electrochem. Soc.* **2005**, *152*, B140-B151.

[S4] D. Barkley, *Phys. Rev. Lett.* **1994**, 72, 164-167.
